# Comparative Transcriptome Analysis of *Streptomyces nodosus* Mutant With a High-Yield Amphotericin B

**DOI:** 10.3389/fbioe.2020.621431

**Published:** 2021-02-01

**Authors:** Kai Huang, Bo Zhang, Yu Chen, Zhi-Qiang Liu, Yu-Guo Zheng

**Affiliations:** ^1^The National and Local Joint Engineering Research Center for Biomanufacturing of Chiral Chemicals, Zhejiang University of Technology, Hangzhou, China; ^2^Key Laboratory of Bioorganic Synthesis of Zhejiang Province, College of Biotechnology and Bioengineering, Zhejiang University of Technology, Hangzhou, China

**Keywords:** *Streptomyces nodosus*, amphotericin, comparative transcriptome, mutagenesis, metabolic pathway

## Abstract

Antibiotics play an important role in human health. Most antibiotics are derived from microbial secondary metabolites. Amphotericin is a polyene macrolide antibiotic synthesized by *Streptomyces nodosus*. *S. nodosus* ZJB2016050 with high-yield amphotericin B (AmB) was obtained by traditional mutagenesis using *S. nodosus* ATCC14899 as the original strain. The differences in the characterization of the two strains were found in color, mycelium morphology, and AmB yield. Subsequent comparative transcriptome explained the yield differences between the two strains. Pathways including the carbohydrate metabolic pathway and the secondary product synthesis pathway were targeted. The upregulation of glucokinase, phosphoglycerate mutase, and pyruvate dehydrogenase accelerates the consumption of glucose and has great effects on the accumulation of precursors. One of the competitive secondary metabolites of the polyketone synthetase (PKS) II type sapromomycin analog synthesis gene cluster was downregulated, which competes for malonyl-CoA. Five PKS modules (except for the first module amphA) of the amphotericin synthetic gene cluster in the high-yielding strain were downregulated, which resulted in the total amphotericin A (AmA) and AmB of *S. nodosus* ZJB2016050 being less than that of the wild-type *S. nodosus* ATCC14899. Combined with gene differential expression in the pentose phosphate pathway and the reaction mechanism of the ER5 domain, the reason that *S. nodosus* ZJB2016050 preferred to synthesize AmB was probably related to intracellular reduction.

## Highlights

- A high-throughput screening method with inhibition zone was constructed to screen amphotericin B high-yield *Streptomyces nodosus*.
- *S. nodosus* ZJB2016050 was obtained by UV and NTG mutagenesis. The yield of AmB was 5.9 g/L, and the by-product AmA was 0.6 g/L in flasks.
- Through comparative transcriptome analysis, the reasons for the high yield of amphotericin B in *S. nodosus* ZJB2016050 were analyzed. The expression difference in the glycolysis pathway, competing secondary metabolites synthetic gene clusters, and the amphotericin synthetic gene cluster are considered to be the reasons for the difference in the AmB yield of the two strains.
- Analysis of the transcriptome showed differences in the transcription levels of *S. nodosus* ZJB2016050 and the wild-type *S. nodosus* ATCC14899. This work provides theoretical support for the subsequent genetic modification of *S. nodosus* ZJB2016050.

## Introduction

Fungal infection is a major global problem with 1.6 million people dead per year (McAlister and Shapiro, 2019). As human immunodeficiency becomes more common, fungal infection is becoming more prevalent and dangerous. Currently, the most effective way to target fungal infections is to use antibiotics. However, the current situation is not optimistic with the increasing antibiotics resistance of pathogenic microorganisms and the lack of novel antibiotic discovery (Amara et al., 2018). The development of new antibiotics and the research of existing antibiotics are still urgent (Li and Vederas, 2009).

Streptomyces species belong to actinomycetes, and more than two-thirds of the antibiotics have been discovered in this genus (Hopwood, 2007). The classes of antifungal agents used for fungal infection include azoles (posaconazole and isavuconazole), polyenes (amphotericin and its lipid formulations), and echinocandins (caspofungin and micafungin) (Nami et al., 2019). Amphotericin B is produced by *Streptomyces nodosus*, which belongs to polyene macrolide antibiotic and is widely used in clinical treatment with good effect (AbuSalah, 1996; Hartsel and Bolard, 1996). AmB is effective against emerging pathogens such as *Candida auris*, a kind of yeast that is multidrug-resistant, is highly infectious, and has high mortality (Escandón et al., 2019; Forsberg et al., 2019). Besides, novel application study of AmB is also popular. The derivative liposomal AmB has the same antibacterial effect but lower nephrotoxicity than AmB-deoxycholate. A new treatment method of AmB in the clinic, such as short-course high-dose treatment, was also developed to improve the curative effect (Jarvis et al., 2019).

Recently, the developments in metabolic engineering, synthetic biology, and bioinformatics have increased the ability to exploit new secondary metabolism or increase the production of secondary metabolites (Brakhage and Schroeckh, 2011; Bilyk and Luzhetskyy, 2016; Ausländer et al., 2017). However, it is still expensive and time-consuming to construct an industrial strain. Besides, a rational design requires the combination of bioinformatics analysis and existing metabolic pathway modification. Except for some model bacteria, most bacteria have limited bioinformatics analysis and tools for genetic modification, such as some unknown gene clusters and a few tools for genetic manipulation of *Streptomyces* (Amara et al., 2018). Traditional mutagenesis methods, such as chemical mutagenesis using *N*-methyl-*N*-nitroso-*N*′-nitrosoguanidine (NTG) or ethyl methanesulfonate (EMS) and physical mutagenesis using ultraviolet (UV) light or X-rays, are still simple and effective methods to generate a high yield of product strains (Baltz, 2016). The efficient screening method is one of the main routes to improve the strain without a comprehensive understanding of various factors affecting the product yield (Parekh et al., 2000; Feng et al., 2019). In addition, combined with the bioinformatics, the analysis of superior mutagenesis strains can explain the mechanism for high yield and provide theoretical guidance for the subsequent improvement of metabolic engineering (Kim et al., 2018).

Gene microarrays are used to obtain the sequence information of mRNA by hybridization between the probe with known sequence and the sample mRNA. If the sample mRNA has never been reported before, there will be no corresponding probe sequence and the new mRNA cannot be detected. In addition, the background noise of nucleic acid hybridization is very high, which affects the accuracy of sequencing. As the high-throughput sequencing technology becomes mature, RNA-sequencing (RNA-seq) is a recently developed approach for transcriptome profiling and is popularly applied in bacteria. RNA-seq technology, which is the reverse transcription of mRNAs into the cDNA library and then sequencing, detects gene expression more sensitively than microarrays. With reference genomes, the genome structural variations and genome-wide gene expression abundance can also be analyzed (Wang et al., 2009).

In this study, *S. nodosus* ATCC14899 was mutated by UV combined with NTG. A strain with high-yield AmB was obtained and named *S. nodosus* ZJB2016050. After observation of the characterization, it was found that the AmB yield, appearance, and color of *S. nodosus* ZJB2016050 were significantly different from those of the wild-type strain. By comparing the transcriptome analysis, the reasons for high yield were discussed. This work can provide a theoretical basis for the metabolic engineering of *S. nodosus*.

## Materials and Methods

### Bacterial Strains, Medium, and Culture

*S. nodosus* ATCC 14899 (*S. nodosus* ZJB20130827) was isolated from soil sample. AmB high-yield mutagenic *S. nodosus* ZJB2016050 (*S. nodosus* N3) was obtained by NTG-UV treatment using an *S. nodosus* ATCC 14899 spore (Zhang et al., 2018). *S. nodosus* strains were cultured in a liquid GYM (glucose yeast extract–malt extract) medium and on a GYM agar medium for culture and sporulation, respectively. Isolation and purification of the mutation were carried out on a GYM agar medium at 28°C for 4–10 days (Kieser et al., 2000).

### UV-Irradiation and NTG Mutagenesis

A total of 10 mL flesh spore suspension was collected and then exposed to 15 W of UV irradiation at 254 nm for 1 min with a 20 cm distance. The spore suspension after UV irradiation was collected to a recovery medium (8 g/L yeast extract, 20 g/L glucose, and 2 g/L peptone) and incubated for 24 h at 28°C. Then, the spores were collected in a 50 mM PBS buffer. One milliliter of the spore suspension was treated with 5 mg/mL NTG for 30 min. The treated spores were spread on a GYM agar medium at 28°C for 6–10 days (Zhang et al., 2018). The colonies that survived were screened using *Saccharomyces cerevisiae* X-33 as the indicator organism. The colonies of positive mutagenesis were verified using high-performance liquid chromatography (HPLC).

### Fermentation, Amphotericin Analysis, and Electron Microscopy of Mycelium

The spore solution was cultivated in a seed medium (15 g/L peptone, 10 g/L glucose, 10 g/L yeast extract, 5 g/L NaCl, and 1 g/L CaCO_3_) and incubated at 25°C shaking at 200 rpm for 2 days. A total of 2 mL fresh hyphae culture was transferred to a fermentation medium (25 g/L cotton-seed meal, 69 g/L glucose, 9 g/L CaCO_3_, and 0.1 g/L KH_2_PO_4_) and incubated at 25°C shaking at 200 rpm for 4 days (Zhang et al., 2018). Amphotericin was extracted by DMSO (dimethylsulfoxide) and shaking at 25°C for 20 min. The mixture was centrifuged at 10,000 rpm for 10 min, and the supernatant was used for HPLC analysis. The HPLC system was an LDC 3200 analytical system (LDC Analytical Inc., New York, USA) with a UV-VIS detector (Yilite Analytical Inc., Dalian, China). The column was an Agilent C18 reversed-phase column (5 μm, 4.6 × 150 mm, Agilent Technologies Inc., Santa Clara, CA, USA), and the mobile phase was methanol/acetonitrile/water (4:7:9/v:v:v). AmA was detected at 304 nm, and AmB was detected at 405 nm. Pure samples of AmB and AmA were purchased from Sigma-Aldrich (St. Louis, MO, USA). The model of the electron microscope is the SU8010 Scanning Electron Microscope (Hitachi, TKY, JPN).

### RNA Isolation and q-PCR

Total RNA isolation of *S. nodosus* ATCC14899 and *S. nodosus* ZJB2016050 was prepared using a Trizol reagent. The *S. nodosus* strains were cultured in a fermentation medium, and the fresh mycelium was collected at 72 h. The mycelium pellets were resuspended with a 200 μL TE buffer (10 mM Tris-HCl, 1 mM EDTA), which contains lysozyme (3 mg/ml) and incubated at 25°C for 5 min. Then 1.5 ml Trizol was added and shaken violently for 3 min, and incubated at 25°C for 5 min. The samples were then centrifuged at 10,000 rpm and 4°C for 5 min. The supernatant was transferred to 2.0 mL EP tubes containing 200 μL of chloroform/isoamyl alcohol. The mixture was vortexed, incubated for 1 min at 25°C, and centrifuged at 10,000 rpm and 4°C for 10 min. After the phase separation, the supernatant was removed into an EP tube. After adding equal volume isopropanol, the mixture was incubated at −20°C for 1 h and then centrifuged at 13,600 rpm and 4°C for 20 min. The supernatant was removed, and the resulting pellets were washed with 75% ethanol and then centrifuged at 10,000 rpm and 4°C for 3 min. The supernatant was removed and the resulting pellets were dried at 37°C for 5 min and resuspended in 100 μL DEPC H_2_O. The samples were treated with DNase I at 37°C for 30 min. The samples were added with 150 μL phenol/chloroform/isoamyl alcohol (25:24:1/v:v:v), then vortexed and centrifuged at 13,600 rpm for 15 min. The aqueous phase was removed into an EP tube and added with 400 μL 0.3 M sodium acetate, then centrifuged at 10,000 rpm and 4°C for 15 min. The resulting sediment was washed twice with 75% ethanol and then dried at 37°C and resuspended in 100 μL DEPC H_2_O. The isolated RNA was stored at −80°C.

The q-PCR was used to verify the accuracy of transcriptome sequencing. The biologically replicated samples are extracted with total RNA. One microgram total RNA of each sample was treated with HiScript Q RT SuperMix for qPCR (Vazyme, NanJing, CHN) to prepare cDNA. ChamQ Universal SYBR qPCR Master Mix (Vazyme, NanJing, CHN) was used for q-PCR process on an ANALYTIKJENA QTOWER real-time fluorescent quantitative PCR instrument (Analytik Jena AG, Jena, GER).

### Transcriptome Library Construction

One microgram of the total RNA sample was treated with a Ribo-Zero™ Magnetic Gold Kit (Illumina, San Diego, CA, USA) to deplete the rRNA, and then the protocol of TruSeq RNA Sample Prep Kit v2 (Illumina, San Diego, CA, USA) was followed to construct the libraries. The first-strand cDNA is generated by First Strand Master Mix and Super Script II (Invitrogen, Carlsbad, CA, USA) reverse transcription with the following reaction condition: 25°C for 10 min; 42°C for 50 min; 70°C for 15 min. The product was purified and added with a Second Strand Master Mix and a dATP, dGTP, dCTP, dUTP mix to synthesize the second-strand cDNA at 16°C for 1 h. The purified fragmented cDNA combine with an End Repair Mix was incubated at 30°C for 30 min. The end-repaired DNA was purified with Ampure XP Beads and added with an A-Tailing Mix, mixed by pipetting and incubated at 37°C for 30 min. The adenylate 3′ends DNA and an RNA Index Adapter and Ligation Mix were combined and mixed well by pipetting, and then incubated at 30°C for 10 min. The end-repaired DNA was purified with Ampure XP Beads and added with the Uracil-N-Glycosylase enzyme. The product was incubated at 37°C for 10 min and purified AGENCOURT Ampure XP Beads. Several rounds of PCR amplification with a PCR Primer Cocktail and a PCR Master Mix are performed to enrich the cDNA fragments. Then the PCR products are purified with Ampure XP Beads. The libraries' quality and quantity are assessed in two methods: by checking the distribution of the fragment size using the Agilent 2100 bioanalyzer instrument (Agilent DNA 1000 Reagents, Agilent Technologies Inc., CA, USA), and by quantifying the library using real-time quantitative PCR (QPCR) (TaqMan Probe, Thermo Fisher Scientific, Waltham, MA, USA). The qualified libraries will amplify on cBot to generate the cluster on the flowcell (TruSeq PE Cluster Kit V3-cBot-HS, Illumina, San Diego, CA, USA). Furthermore, the amplified flowcell will be sequenced on the HiSeq 2000 System (TruSeq SBS KIT-HS V3, Illumina); a read length of 90 is the most common sequencing strategy.

### Bioinformatics Analysis of Sequencing Reads

The sequenced raw reads were filtered to get clean reads and mapped to the *S. nodosus* ATCC14899 genome (Genebank ID: NZ_CP009313) using HISAT v2.0.1-beta (Kim et al., 2015). The clean reads were aligned to the reference gene using Bowtie (http://bowtie-bio.sourceforge.net/Bowtie2/index.shtml), and the gene expression levels were calculated using RSEM (http://deweylab.biostat.wisc.edu/RSEM). The screening of differentially expressed genes was analyzed by the PossionDis method in this study because it allows samples with no replicates (Audic and Claverie, 1997).

$$\begin{array}{l} \text{p}\left(\text{x}\right)=\frac{{\text{e}}^{-\lambda \;}{\lambda \;}^{\text{x}}\;}{\text{X}!} \end{array}$$

X refers to the reads number of the corresponding gene. The screening criteria for significantly differentially expressed genes were abs log_2_ (FoldChange) ≥ 1 and FDR ≤ 0.005.

The pathway significant enrichment analysis refers to the KEGG (Kyoto Encyclopedia of Genes and Genomes) pathway database in this study.

$$\begin{array}{l} \text{P}=1-\overset{m-1}{\underset{i=0}{\sum }}\frac{\left(\begin{array}{c} \text{M}\\ i \end{array}\right)\left(\begin{array}{c} \text{N}-\text{M}\\ n-i \end{array}\right)}{\left(\begin{array}{c} \text{N}\\ n \end{array}\right)} \end{array}$$

N refers to the number of genes with pathway annotation in all genes. *n* is the number of differentially expressed genes (or target genes) in N. M refers to the number of genes annotated as a specific pathway in all genes. *m* is the number of significantly differentially expressed genes (or target genes) noted in a particular pathway. A pathway with *Q* ≤ 0.05 was defined as that was significantly enriched in significantly differentially expressed genes. Through pathway significant enrichment, the significantly differentially expressed genes of major metabolic pathways and signal transduction pathways can be identified.

The GO (Gene Ontology) function enrichment analysis refers to the Gene Ontology database (http://www.geneontology.org/) using the GO Termfinder (http://www.yeastgenome.org/help/analyze/go-term-finder). The calculation formula of GO enrichment analysis is similar to that of KEGG enrichment. A GO term with *P* ≤ 0.05 was defined as a GO term that was significantly enriched in significantly different genes. The GO functional significance enrichment analysis was used to determine the biological functions of the significantly different genes.

## Results

### Mutagenesis Results and Morphological Observation

The spore morphology of the mutant strains was classified into three types. The mutant strains of type 1 had no spores, and the color was yellowish. The shape of the colony on the agar medium was deplanate. This kind of mutant colonies may have some internal dysfunction, although not fatal; the rate of growth and spore formation have been affected. The overall level of growth was slower than the original strain, and the positive mutation rate was low. The color of the type 2 mutant strains was yellow, and the shape was convex. The type 2 colonies generated black spores, and the positive mutation rate was 15.8%, which is higher than that of type 1. The type 3 mutant was the best kind of mutant strains with a positive mutation rate of 72.6%. The color of the colonies was yellowish, and the shape was convex. The type 3 mutant generated the common spores, which became gray at 2–3 days. The bulge in colony morphology means that the mutant strains of the latter two types have little effect on the growth rate, which is similar to the original strain *S. nodosus* ATCC14899, while the difference in the spore color is likely related to the intracellular pigment, which is likely related to the mass of the spores. Among the three types of mutagenesis, the AmB production of types 1 and 2 mutagenesis clones was not significant, and the yield was about the same as the original strain. The high-yield mutant *S. nodosus* ZJB2016050 from type 3 mutagenesis was screening out for culture. The seed liquid of *S. nodosus* ZJB2016050 was reddish, and the mycelium was granular. In contrast, the seed solution of *S. nodosus* ATCC14899 was yellow, and the shape is globular (Supplementary Figure 1). The agar diffusion assay screening method is shown in Supplementary Figure 2. The characteristic and the positive mutant rate were listed in Supplementary Table 1.

### Electron Microscopy and Fermentation Analysis

**Figure 1:**
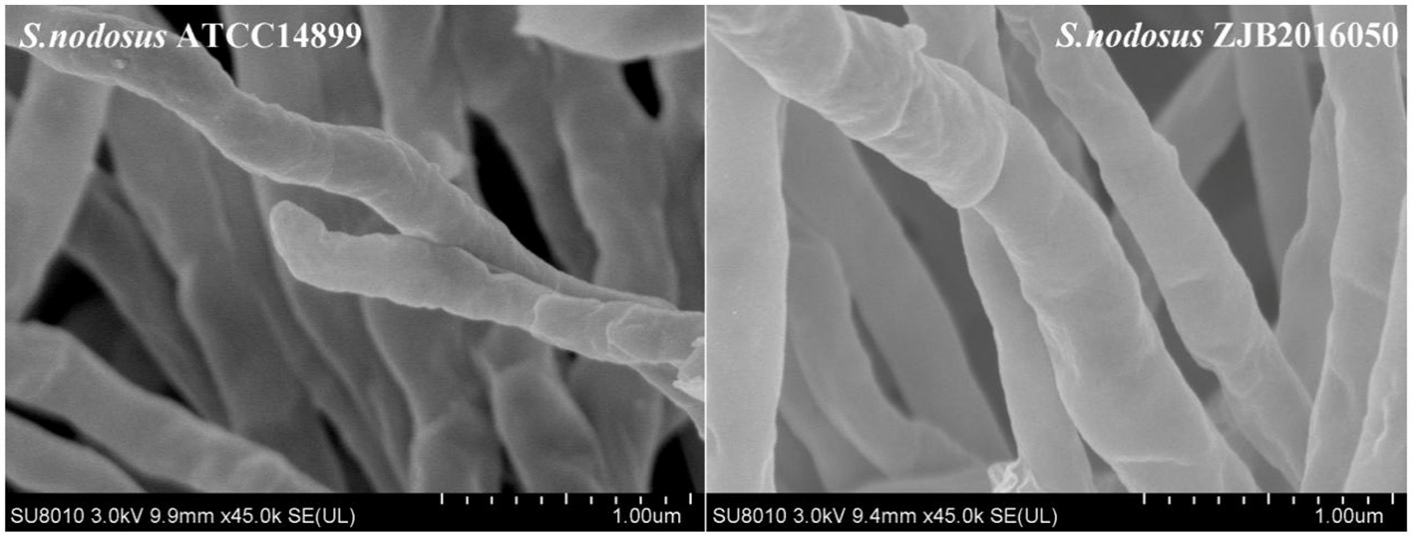
Electron micrographs of the mycelium of *S. nodosus* ATCC14899 and *S. nodosus* ZJB2016050; the minimum scale was 1 μm.

The result of electron microscope screening for *S. nodosus* ZJB2016050 and *S. nodosus* ATCC14899 is shown in Figure 1. At a magnification of 45,000×, the mycelium of *S. nodosus* ZJB2016050 was found to be significantly thicker than the mycelium of *S. nodosus* ATCC14899.

**Figure 2:**
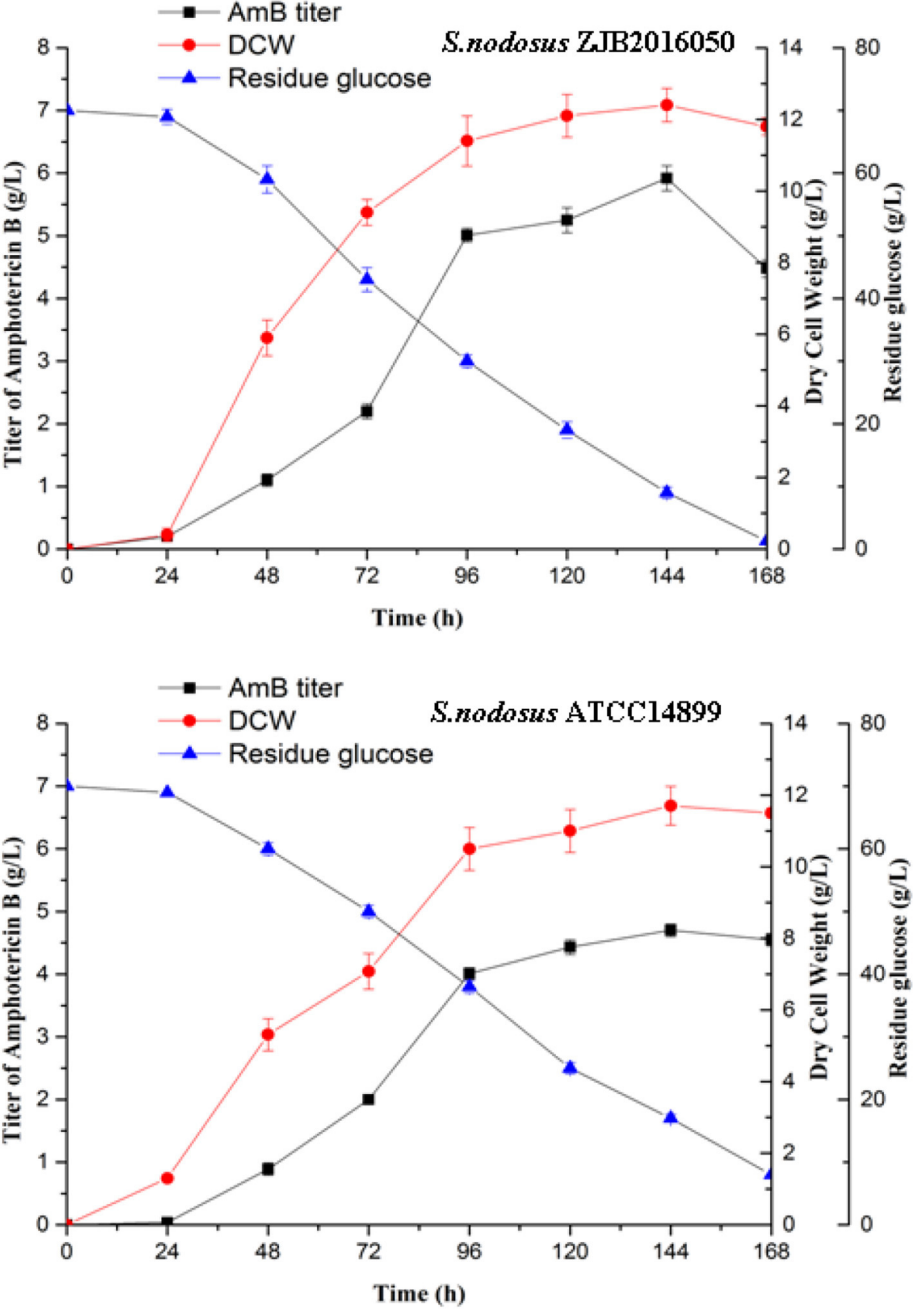
The shake flask fermentation process of *S. nodosus* ATCC14899 and *S. nodosus* ZJB2016050. Black squares represent AmB titer, red circles represent DCW, and blue triangles represent residue glucose.

The mutation *S. nodosus* ZJB2016050 and the origin *S. nodosus* ATCC14899 were fermented in a shaking flask, and the parameters including AmB yield, dry cell weight, and residue glucose were compared (Figure 2). The mutant strain had some advantages in the growth and had a longer exponential growth period than the wild type. Both strains grew most rapidly in the 24–96 h period. The cotton-seed meal is in the form of granules, which is helpful to reduce clumping during the fermentation of *S. nodosus*. Both of the strains growth and the amphotericin synthesis required glucose. The detection of residue glucose accurately reflected the ability of *S. nodosus*. From the fermentation process curve, the glucose consumption rate of the high-yield *S. nodosus* ZJB2016050 was accelerated. The glucose was almost consumed at 168 h, while the residue glucose of the origin *S. nodosus* ATCC14899 was 7.9 g/L. The accumulation of amphotericin was accompanied by the increase in biomass and seemed to be related to the biomass. The AmB yield of *S. nodosus* ZJB2016050 peaked at 144 h and declined thereafter, which was due to the low glucose concentration leading to nutrient deficiency. The yield of by-product AmA was tested at 96 h. The AmA and AmB fermentation yields of *S. nodosus* ZJB2016050 were 0.6 and 5.9 g/L, respectively, while the yields of *S. nodosus* ATCC14899 were 3.3 and 4.7 g/L, respectively (Supplementary Figure 3). Due to the similar structure and the similar biosynthesis pathways, it often takes more effort to remove AmA in industrial production. Based on the fermentation advantage of *S. nodosus* ZJB2016050, it is meaningful to compare transcriptome analysis in this study.

### Analysis of the Transcriptome Reads of *S. nodosus*

The poor accuracy reads will affect the subsequent quantitative analysis, and the reads with a high content unknown base also interfere with the analysis of the reliability (Li et al., 2008; Cock et al., 2009). To ensure the quality of the information analysis, the original data in low quality, adapter pollution and the reads with high content unknown base were removed before the data analysis. A total of 8.77 and 8.83 Mb clean reads were obtained from *S. nodosus* ZJB2016050 and *S. nodosus* ATCC14899, respectively. The percentage of low-quality reads of *S. nodosus* ZJB2016050 and *S. nodosus* ATCC14899 were 15.35 and 18.11%, respectively. The quality of filtered reads is summarized in Table 1. The distribution of the base content of each sample is shown in Supplementary Figure 4. The base mass distribution of each sample is shown in Supplementary Figure 5. The composition profile of total raw reads is shown in Supplementary Figure 6.

**Table 1:** The quality summary of filtered reads.

**Sample**	**Total raw reads (Mb)**	**Total clean reads (Mb)**	**Total clean based (Gb)**	**Clean reads Q30 (%)**	**Low quality reads (%)**	**Adapter reads (%)**	**GC content**
ZJB2016050	11.43	8.77	1.31	90.76	15.35	7.92	67.25
ATCC14899	11.43	8.83	1.32	90.40	18.11	4.63	68.65

*Q30 reads mean a mass value >30, which indicates a base error rate of 0.1%*.

**Figure 3:**
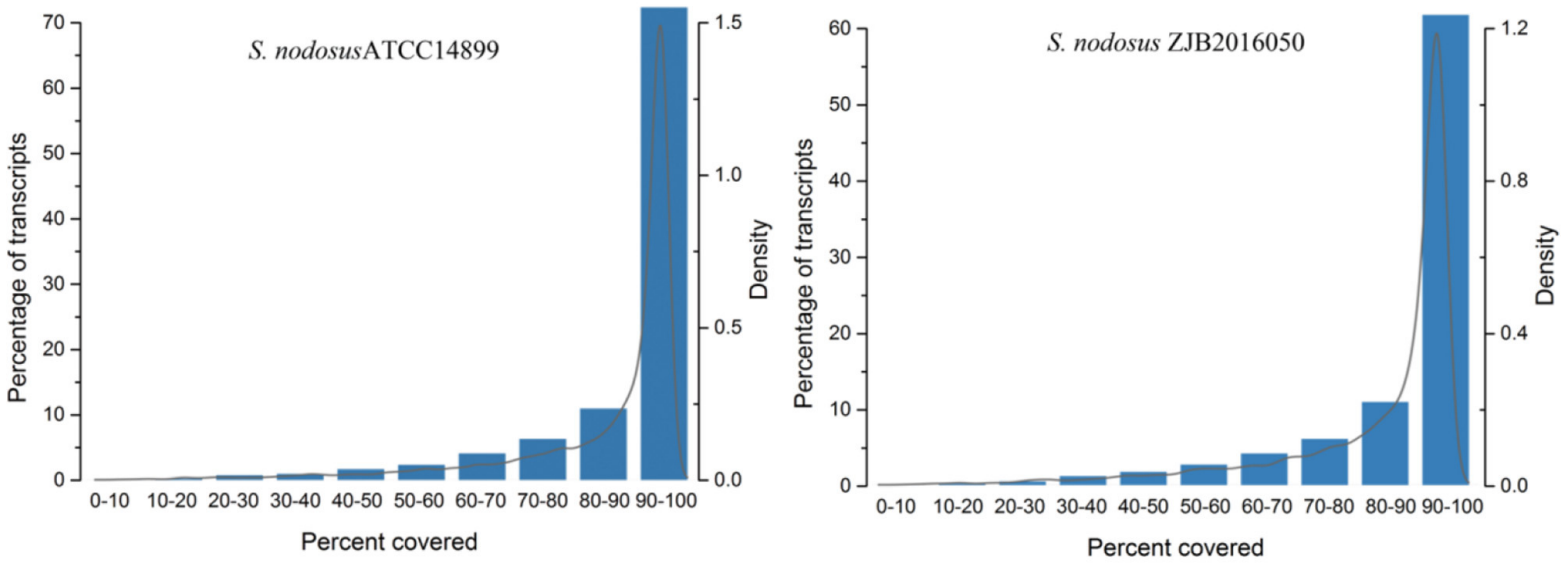
The genes coverage of samples of *S. nodosus* ATCC14899 and *S. nodosus* ZJB2016050.

The comparison rate with reference genome is one of the important indexes to evaluate transcriptome sequencing data. The comparison ratio of each sample was above 95%. The unique match rate of *S. nodosus* ZJB2016050 and *S. nodosus* ATCC14899 was 95.30 and 93.59%, respectively. Normally, sequencing reads evenly covered all positions of the whole length of the genome; however, the distribution of reads was affected by sample degradation, RNA fragmentation bias, and PCR amplification bias. The homogeneity of sequencing results is shown in Supplementary Figure 7, which displays the reads distribution in the 5′-3′ regions of all the genes of the samples. There was no obvious peak in Supplementary Figure 7, which indicates that the sequencing results are relatively uniform. The gene coverage of each sample is shown in Figure 3. As the peak value in the figure was on the right, it indicates that the gene coverage was good; otherwise, there may be insufficient data or bias. For samples with good quality and enough sequencing data, most of the genes would be fully covered.

### Gene Expression Pattern Analysis for *S. nodosus*

**Figure 4:**
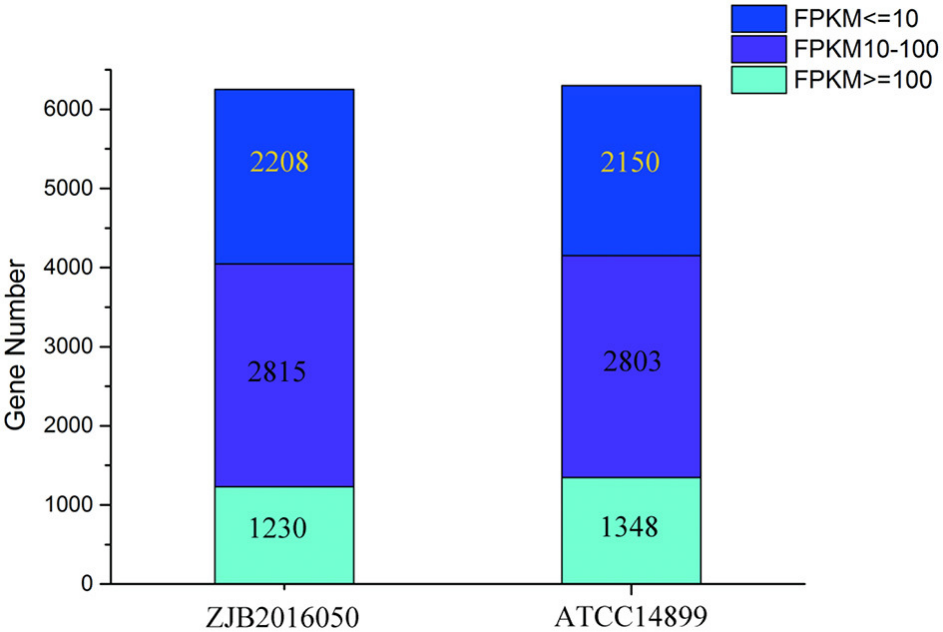
The number distribution of genes with different expression levels in samples of *S. nodosus* ATCC14899 and *S. nodosus* ZJB2016050. Light blue represents genes with FPKM ≥ 100, purple represents genes with FPKM 10–100, and dark blue represents genes with FPKM ≤ 10.

The number distribution of genes with different expression levels in each sample is shown in Figure 4. The value of fragments per kilobase million mapped reads (FPKM) is represented for the gene expression. Among the expression genes, a total of 6,103 genes were common genes, and 149 and 193 genes were the number of unique genes for *S. nodosus* ZJB2016050 and *S. nodosus* ATCC14899, respectively (Supplementary Figure 8). If there are significant phenotypic or functional differences between the two strains, it is usually due to the differences in gene expression between the two samples. Therefore, the comparative analysis of differentially expressed genes is often the most important content in comparative transcriptome. Among the difference expression genes (DEGs), 414 genes of *S. nodosus* ZJB2016050 were upregulated and 660 genes were downregulated (Supplementary Figure 9).

**Figure 5:**
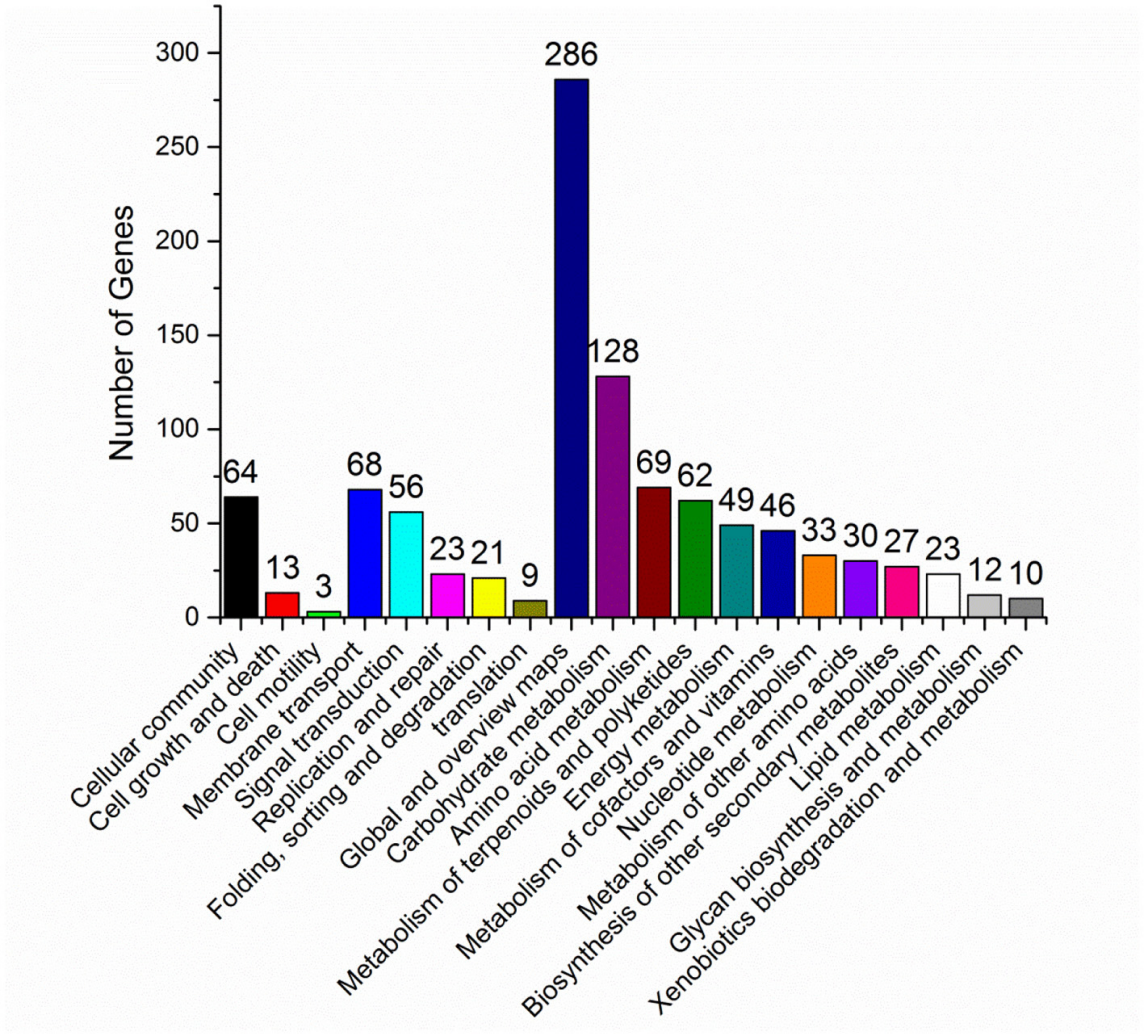
KEGG pathway classification map of significantly differentially expressed genes.

*In vivo*, different genes coordinate their biological functions. Pathway-based analysis could identify the major biochemical pathways and signal transduction pathways with significantly DEGs. Among the significantly DEGs between *S. nodosus* ZJB2016050 and *S. nodosus* ATCC14899, the metabolic classification with the largest number of DEGs was global metabolism, and carbohydrate metabolism also had large different expressed genes with 123 genes in total (Figure 5). In addition, the number of DEGs associated with polyketides metabolism was 68, which was also a relatively large number of differentially expressed genes. Amphotericin is one of the polyketides, and the potential reasons for the yield differences may lie in these DEGs. About 11 genes are involved in cell growth and death, which is a relatively small number.

**Figure 6:**
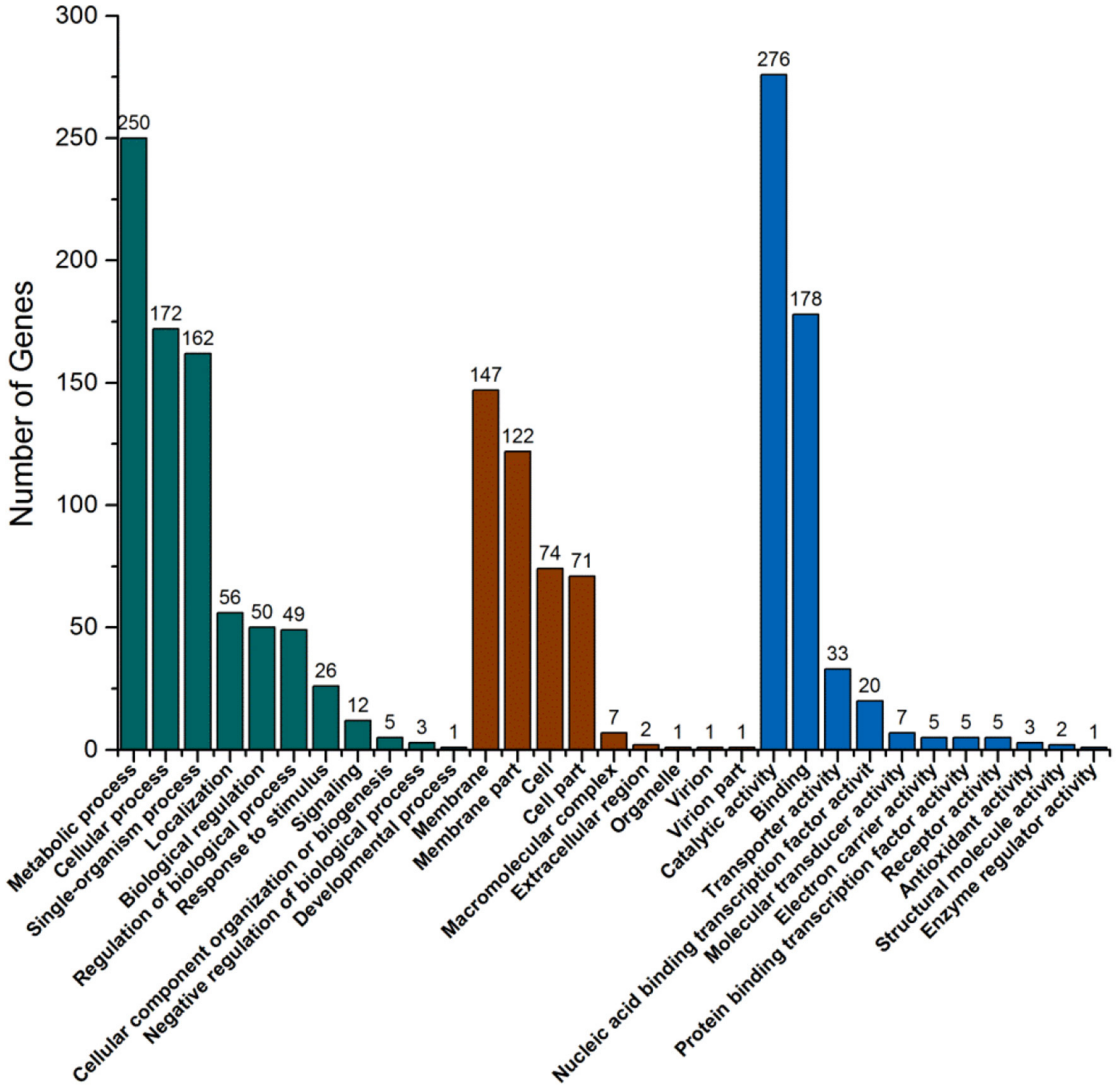
GO function classification map of significantly differentially expressed genes.

There are three ontologies in GO that describe the molecular function, cell component, and biological process of genes, respectively. GO function enrichment analysis gives the GO function items that are enriched in the significantly DEGs compared with the genomic background to show which biological functions are correlated with the significantly DEGs. In biological process ontology, the most different item was metabolic process with 375 DEGs, and cellular process was the second most different item with 223 DEGs (Figure 6). These large differences are probably the main reason for the growth differences between strains. In cell component ontology, membrane and membrane part items had significant differences with 201 and 192 DEGs, respectively. In molecular function ontology, the most different item was catalytic activity with 403 DEGs. This may indicate that the pathway with these DEGs is likely to be regulated. Combined with the KEGG pathway and GO analysis, the functional localization of DEGs was relatively accurate. This can help to provide reliable bases for speculating about the causes of the strain differences.

### Carbohydrate Metabolic Pathway Analysis

Carbohydrate metabolism is one of the main metabolic pathways and energy supply in bacteria, including the precursor supplying of polyketides synthesis, intracellular reductive-power supplying, energy metabolism, etc. (Yang et al., 2013; Kwak and Jin, 2017; Liu et al., 2019). The fermentation of amphotericin takes glucose as the only carbon source in this study, and it is necessary to analyze the carbohydrate metabolism pathway.

**Figure 7:**
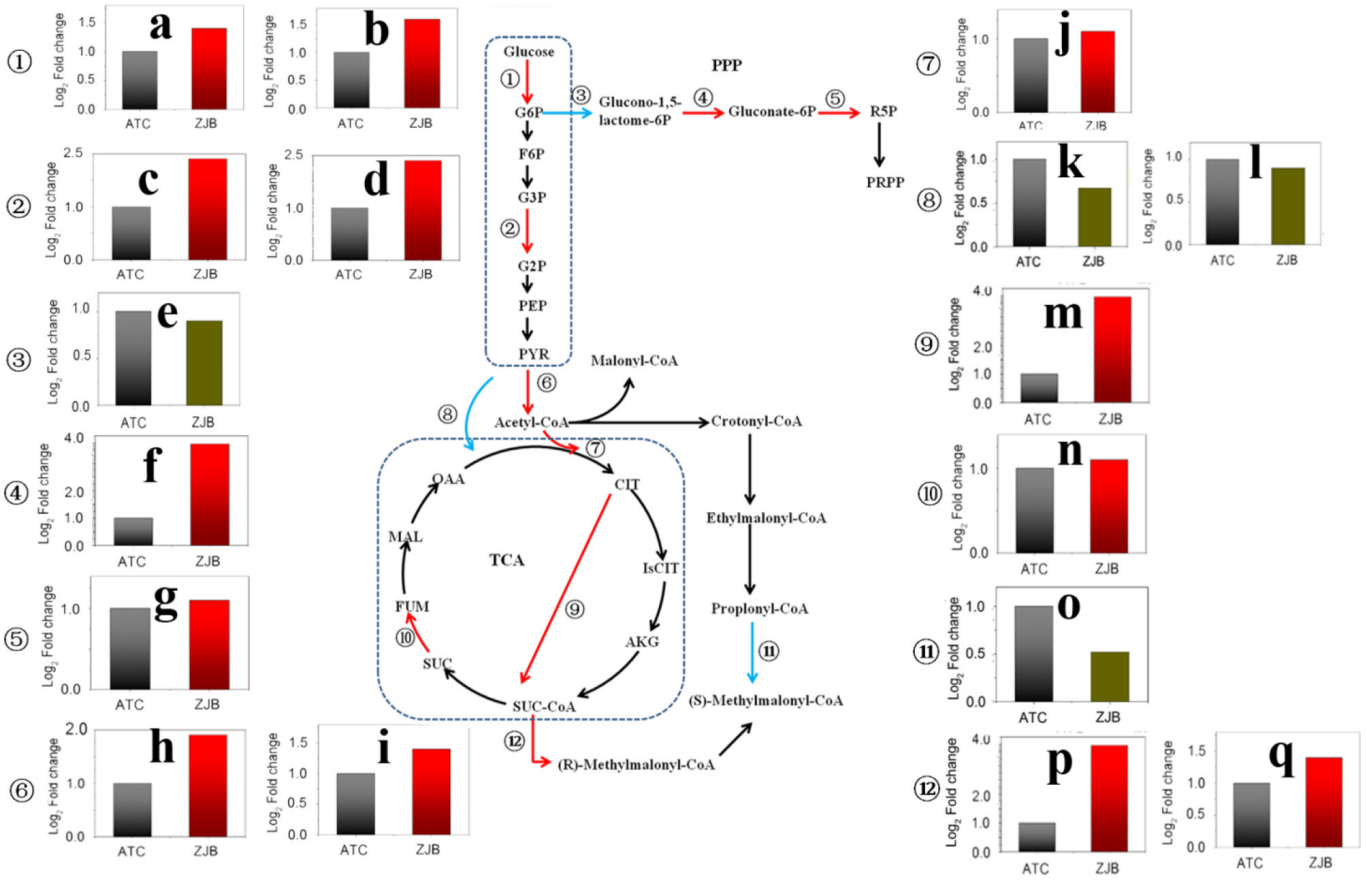
Significantly differentially expressed genes in important metabolic pathways, including the glycolysis pathway, the pentose phosphate pathway, the TCA cycle, and the amphotericin precursors synthesis. The red arrow in the metabolic pathway diagram represents gene upregulation. The blue arrow represents gene downregulation. The fold difference in gene expression is presented in histograms. The red column represents gene upregulation, and the dark green column represents gene downregulation. **(a)** SNOD_RS32555 sugar kinase. **(b)** SNOD_RS25490 sugar kinase. **(c)** SNOD_RS24025 phosphoglycerate mutase. **(d)** SNOD_RS06850 phosphoglycerate mutase. **(e)** SNOD_RS27415 glucose-6-phosphate dehydrogenase. **(f)** SNOD_RS10480 O-methyltransferase. **(g)** SNOD_RS16575 6-phosphogluconate dehydrogenase. **(h)** SNOD_RS16815 pyruvate dehydrogenase. **(i)** SNOD_RS16810 pyruvate dehydrogenase. **(j)** SNOD_RS14500 citrate synthase. **(k)** SNOD_RS29275 TetR/AcrR family transcriptional regulator. **(l)** SNOD_RS26215 pyruvate carboxylase. **(m)** Unlabeled gene2 2-oxoglutarate dehydrogenase. **(n)** SNOD_RS04655 succinate dehydrogenase. **(o)** SNOD_RS20250 propionyl-CoA carboxylase. **(p)** SNOD_RS22165 methylmalonyl-CoA epimerase. **(q)** SNOD_RS26855 methylmalonyl-CoA mutase.

The pathway from glucose to acetyl-CoA was significantly upregulated at three nodes, including glucose to D-glucose-6P (G6P), glycerate-3P (G3P) to glycerate-2P (G2P), and pyruvate to acetyl-CoA. The expression of *SNOD*_RS25490 and *SNOD*_RS32555 genes, which were identified as sugar kinase and participated in the step from glucose to G6P, had an increase of 1.6- and 1.4-fold (log2 FPKM/FPKM). *SNOD*_RS24025 and *SNOD*_RS06850 genes were identified as phosphoglycerate mutase participating in step G3P to G2P with an increase of 1.9- and 2.0-fold (log_2_ FPKM/FPKM), respectively. *SNOD*_RS16815 and *SNOD*_RS16810 were identified as pyruvate dehydrogenase and increased 1.7- and 1.2-fold (log_2_ FPKM/FPKM), respectively (Figure 7).

As acetyl-CoA enters the tricarboxylic acid cycle (TCA), the expression of *SNOD*_RS14500 gene (citrate synthase) and unlabeled gene 2 (2-oxoglutarate dehydrogenase) was upregulated by 1.1-fold (log_2_ FPKM/FPKM) and 3.8-fold (log_2_ FPKM/FPKM), respectively. The 2-oxoglutarate dehydrogenase participated in step from 2-oxoglutarate to succinyl-dihydrolipoamide. This could lead to increased accumulation of succinyl-CoA. Besides, the expressions of methylmalonyl-CoA epimerase (*SNOD*_RS22165) and methylmalonyl-CoA mutase (*SNOD*_RS26855) in the pathway from succinyl-CoA to methylmalonyl-CoA were increased. In contrast, the amount of propionyl-CoA that flows to methylmalonyl-CoA may be reduced due to a 2.1-fold (log_2_ FPKM/FPKM) reduction for propionyl-CoA carboxylase (*SNOD*_RS20250) (Figure 7).

The pentose phosphate pathway (PPP) is the main metabolic pathway in which bacteria acquire NADPH in the cell. Two genes with significantly different expression were glucose-6-phosphate dehydrogenase (*SNOD*_RS27415) and 6-phosphogluconate dehydrogenase (*SNOD*_RS16575), and both of them are on metabolic catalytic reaction to produce NADPH (Figure 7). In the step from G6P to glucono-1,5-lactone-6P, *SNOD*_RS27415 gene was downregulated by one-fold (log_2_ FPKM/FPKM). In the step from D-gluconate-6P to D-ribulose-5P, the *SNOD*_RS16575 gene was upregulated by 1.1-fold (log_2_ FPKM/FPKM).

### Multiple Genes or Metabolic Pathways Analysis

#### The Most Significant Difference Expressions Attributive Metabolic Pathway

Among the significant gene expression differences between high-yielding strains and wild-type strains, the first 20 upregulated and downregulated genes with the greatest difference were selected (Table 2). In the *S. nodosus* database, the functions of multiple genes or proteins have not been identified, and a total of 18 genes were considered as hypothetical protein.

**Table 2:** List of the top 20 genes of *S. nodosus* ZJB2016060 with the most differences in upregulated genes and downregulated genes, respectively.

**Gene tag**	**Gene length**	**Product**	**Log_**2**_Fold change**
Unlabeled gene 3	189	Hypothetical protein	14.00
SNOD_RS08105	186	Hypothetical protein	12.74
SNOD_RS33220	297	Ferredoxin	12.16
SNOD_RS27215	426	Serine/threonine protein kinase	11.94
SNOD_RS25090	588	Serine/threonine protein kinase	11.38
SNOD_RS29130	333	Hypothetical protein	11.34
SNOD_RS19855	579	RNA polymerase	10.08
SNOD_RS32210	822	Hypothetical protein	9.64
Unlabeled gene 4	1,293	Transposase	9.21
SNOD_RS32200	1,464	Hypothetical protein	8.28
SNOD_RS33715	1,992	Hypothetical protein	8.06
SNOD_RS26455	936	Hypothetical protein	7.78
SNOD_RS26450	1,896	Hypothetical protein	7.32
SNOD_RS14330	942	30S ribosomal protein S6 modification protein RimK	7.31
SNOD_RS19985	873	Phosphate ABC transporter ATP-binding protein	7.09
SNOD_RS19980	909	Hypothetical protein	6.59
SNOD_RS14335	312	Hypothetical protein	6.38
SNOD_RS19960	1,758	Phosphate ABC transporter substrate-binding protein	6.07
SNOD_RS19965	2,715	Hypothetical protein	5.97
SNOD_RS19950	990	Phosphate ABC transporter permease subunit PstC	5.55
SNOD_RS26380	1,149	Dehydrogenase	−11.86
SNOD_RS10270	312	Hypothetical protein	−11.78
SNOD_RS34580	537	Hypothetical protein	−11.58
SNOD_RS30730	570	ATP-binding protein	−11.56
SNOD_RS03230	510	Hypothetical protein	−10.48
SNOD_RS30745	1,185	FAD-dependent oxidoreductase	−10.27
SNOD_RS28195	969	Hypothetical protein	−9.29
SNOD_RS32835	1047	Iron ABC transporter	−9.16
Unlabeled gene 5	1,494	α,α-trehalose-phosphate synthase	−6.07
SNOD_RS11045	1,122	FAD-dependent oxidoreductase	−5.96
Unlabeled gene 6	1,734	Trehalose synthase	−5.64
SNOD_RS10530	456	DoxX family protein	−5.56
Unlabeled gene 7	885	dTDP-4-dehydrorhamnose reductase	−5.50
SNOD_RS03660	432	Protease inhibitor protein	−5.06
Unlabeled gene 8	1,056	Sugar kinase	−5.00
SNOD_RS19005	888	Hypothetical protein	−4.61
SNOD_RS33580	2,202	Hypothetical protein	−4.52
SNOD_RS30950	639	ATP-dependent Clp protease proteolytic subunit	−4.41
Unlabeled gene 9	1,212	Aminobutyrate aminotransferase	−4.30
SNOD_RS30740	2,535	Hypothetical protein	−4.18

Among the upregulated genes, *SNOD*_RS33220 was ferredoxin, which participates in redox chains and plays an important role in electron transfer processes and various enzymatic reactions (Ta and Vickery, 1992). Both *SNOD*_RS27215 and *SNOD*_RS25090 were considered as serine/threonine protein kinase, and *SNOD*_RS27215 belongs to the HATPase superfamily, which works as signal transduction (Dufour and Haldenwang, 1994). *SNOD*_RS32210 was putative NitT/TauT family transport system permease protein that helps *S. nodosus* to transport nitrate (Omata et al., 1999). *SNOD*_RS19985, *SNOD*_RS19960, and *SNOD*_RS19950 were considered as phosphate ABC transporters belonging to the ATPase component, the periplasmic component, and the permease component, respectively (Tam and Saier, 1993; Wanner, 1993; Tomii and Kanehisa, 1998).

Among the downregulated genes, *SNOD*_RS26380 was considered as myoinositol,2-dehydrogenase in streptomycin biosynthesis. The synthesis of streptomycin from D-glucose-6P of the glycolysis pathway has a competitive effect on glucose flow to other pathways (Flatt and Mahmud, 2007). *SNOD*_RS30745 and *SNOD*_RS11045 belong to FAD-dependent oxidoreductase and are involved in the halogenation of other polyketides and the mono-oxidation of secondary metabolites. Unlabeled gene 8 considered as sugar kinase was participating in the biosynthesis of other secondary metabolites (the synthetic cluster is partially similar to validamycin).

### Difference Expression in Metabolic Pathways of Secondary Metabolites

The mapping of secondary metabolite synthesis gene clusters was performed with antiSMASH (https://antismash.secondarymetabolites.org) (Medema et al., 2011). Synthesis gene clusters with more than 80% similarity were analyzed (Table 3). The transcriptome expression level of the synthesis gene of the lassopeptide type product MS-271, the siderophore type product desferrioxamine B analog, the ectoine type product ectoine, and the NRPS (non-ribosomal peptide synthetase) type product coelichelin seemed to be low.

**Table 3:** List of secondary metabolites with similarities higher than 80% synthesize gene clusters in *S. nodosus* using antiSMASH.

**Location of genome**	**Type**	**Most similar product**	**Similarity (%)**
130,513–153,027	Lassopeptide	MS-271	100
173,085–223,697	NRPS	Coelichelin	100
1,107,947–1,230,950	Type II PKS-Type III PKS-NRPS	Sapromomycin	86
2,000,465–2,030,585	Terpene	Geosmin	100
2,903,942–2,924,994	Terpene	Albaflavenone	100
4,319,873–4,392,382	Type II PKS	Spore pigment	83
5,011,409–5,023,199	Siderophore	Desferrioxamine B	83
5,895,139–5,905,534	Ectoine	Ectoine	100
7,170,189–7,317,853	Type I PKS	Amphotericin	100

**Figure 8:**
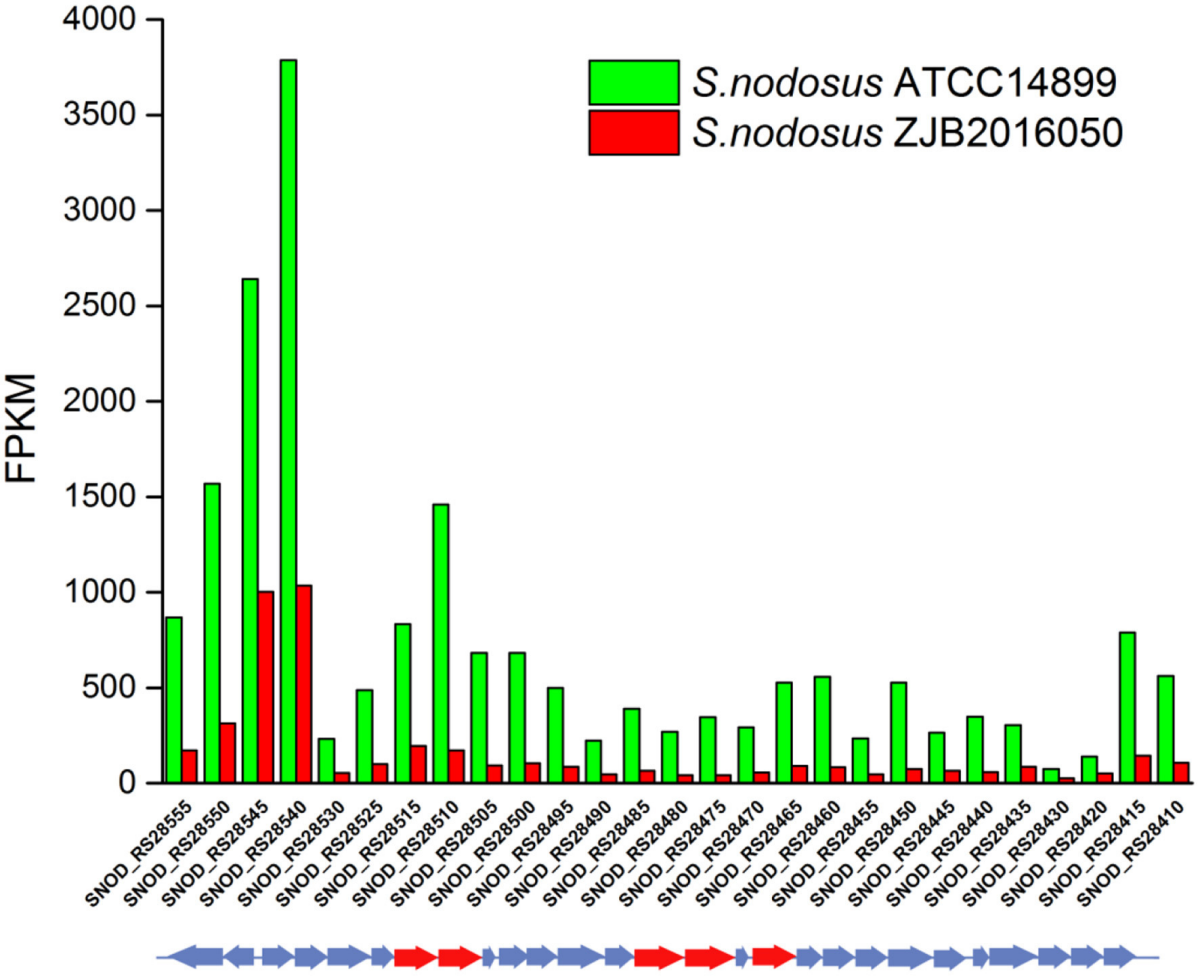
The transcription comparison of the type II polyketone synthetase (PKS5) of the sapromomycin analog synthetic gene cluster of *S. nodosus*. Green bars indicate sample *S. nodosus* ATCC14899, and red bars indicate sample *S. nodosus* ZJB2016050. The red arrows represent the core genes of the synthetic gene cluster.

An 86% similarity sapromomycin product was identified to be a combination of types. The type II polyketone synthetase (PKS5) of the sapromomycin analog synthesis gene is considered as a competing branch of type I PKS because it will compete with the synthetic precursor with type I PKS. The type II polyketone synthetase is highly expressed in *S. nodosus*. By comparison, sapromomycin analog synthesis genes were downregulated in high-yield *S. nodosus* ZJB2016050 (Figure 8). Based on the metabolic pathway prediction of the KEGG pathway, it was found that the core genes *SNOD*_RS28520 and *SNOD*_RS28515 could use malonyl-CoA to synthesize the skeleton of competing products. This may be one of the important reasons for the high yield.

**Figure 9:**
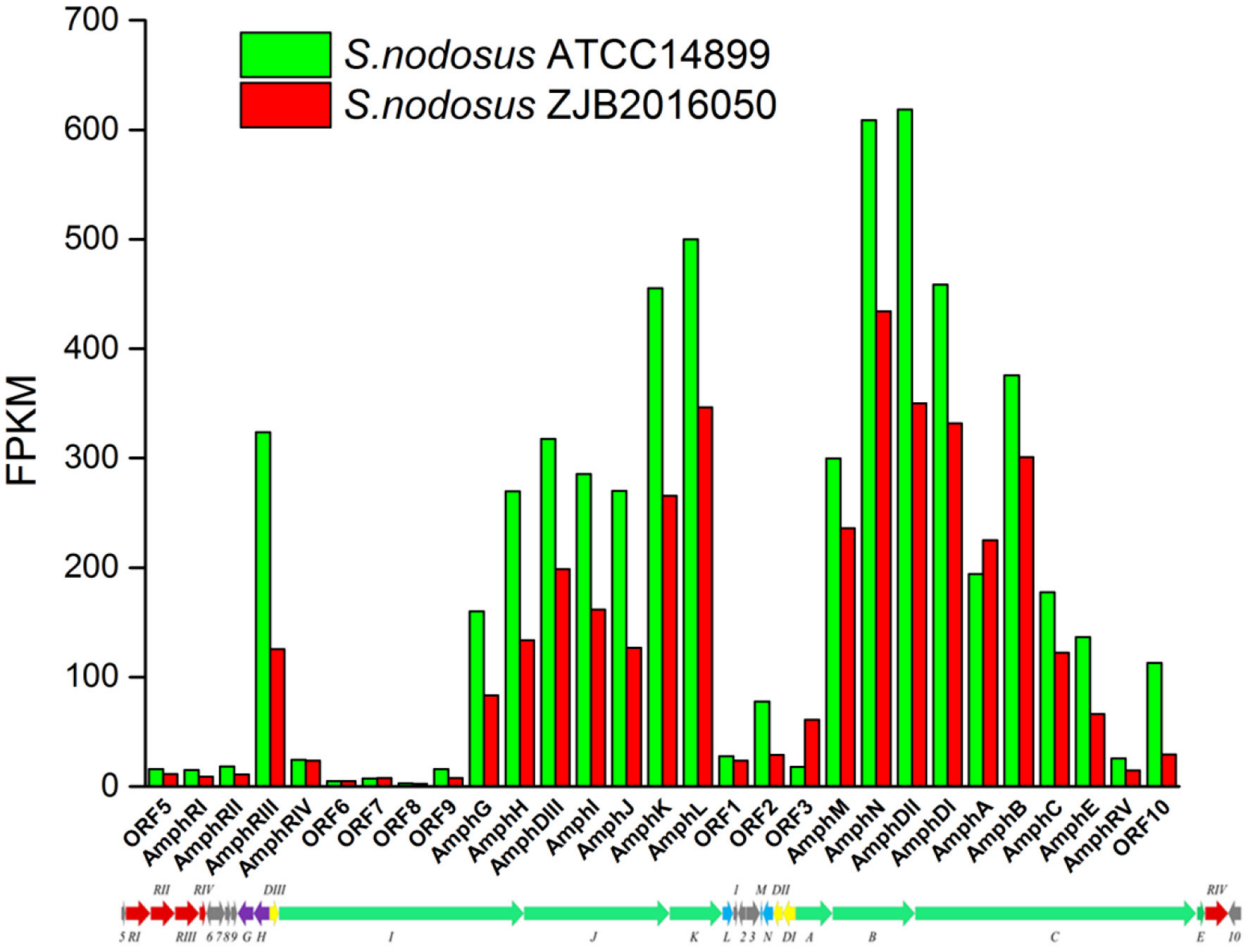
The transcription comparison of the amphotericin biosynthetic gene cluster of *S. nodosus*. Green bars indicate sample *S. nodosus* ATCC14899, and red bars indicate sample *S. nodosus* ZJB2016050. The green arrows represent the core genes of the synthetic gene cluster. The red arrows represent the regulatory factors of the amphotericin synthetic gene cluster. The purple arrows represent transporters of the synthetic gene cluster.

The synthetic gene cluster of amphotericin was reported (Caffrey et al., 2001). In general, the high-yield *S. nodosus* ZJB2016050 was slightly downregulated on the amphotericin synthesis gene cluster compared with the wild-type *S. nodosus* ATCC14899 (Figure 9). The most downregulated gene was *SNOD*_RS02475, a hypothetical protein defined as ORF2 in amphotericin synthesis whose function is unknown. In high-yield *S. nodosus* ZJB2016050, the expression of the amphA module increased at 0.2-fold (log_2_ FPKM/FPKM). However, except for the first start module amphA, all the other PKS modules were shown to be slightly downregulated. The amphK module was downregulated significantly, which is the end PKS module of these six PKS modules.

The q-PCR verification of the 25 key genes in this study is shown in Supplementary Figure 10, which involved the glycolysis pathway, the TCA cycle, the PPP pathway, the competitive secondary metabolite (PKS5) synthesis gene cluster, and the amphotericin synthesis gene cluster.

## Discussion

Traditional mutagenesis is one of the main methods for constructing excellent strains, and rapid screening methods can improve the efficiency of screening. In the screening process of mutagenesis, *S. nodosus* with good spore growth are more likely to have excellent mutant strains. We speculate that the excellent morphology of the spores also shows, to a certain extent, whether the mutant strain has good genetic stability. In this study, a mutated high-yield strain *S. nodosus* ZJB2016050 was obtained in our lab and had greater advantages in industrial production. By comparing the fermentation parameters, it was found that the mutant *S. nodosus* ZJB2016050 had an increased ability to absorb nutrients or increased the nutrients allocated to the growth of the strain. At the same time, we also speculate that this may be due to dense hyphae or rapid hyphae growth. Even without glass beads, *S. nodosus* ZJB2016050 will not form pellets in the liquid medium during fermentation, which may help to improve the utilization of nutrients and has special significance for the fermentation of actinomycetes.

By comparative transcriptome analysis, a large number of differences exist in carbohydrate metabolism, which may explain why high-yielding strain used glucose more rapidly. The expression of glucokinase and phosphoglycerate mutase was upregulated and promoted the utilization of glucose. These steps accelerate the consumption of glucose in fermentation. Upregulation of pyruvate dehydrogenase and downregulation of aldehyde dehydrogenase contribute to the accumulation of acetyl-CoA. In addition to being one of the precursors of amphotericin, acetyl-CoA is also an important up-stream precursor of other metabolic pathways. It was speculated that the glucose consumption rate of the mutagenesis *S. nodosus* was faster than that of the wild type, which would accelerate the synthesis of secondary metabolites and make mycelium grow strong. Besides, robust mycelia could accumulate more secondary metabolites.

Expression differences of genes in the TCA cycle play a positive role in the accumulation of succinyl-CoA. With the upregulation of methylmalonyl-CoA mutase and methylmalonyl-CoA epimerase and the downregulation of propionyl-CoA carboxylase, the accumulation of methylmalonyl-CoA as one of the synthesis precursors may be increased. In another study, supplying the precursor methylmalonyl-CoA will help in AmB biosynthesis (Huang et al., 2020).

A type II PKS(PKS5) of sapromomycin analog synthesis gene clusters is downregulated in *S. nodosus* ZJB2016050. Competing secondary metabolites are more common in *Streptomyces*. In *S. nodosus*, the sapromomycin analog will compete with malonyl-CoA, which is the most important synthetic precursor for the synthesis of amphotericin. In another study, amphotericin production increased by knocking out these genes in *S. nodosus* ZJB2016050 (Huang et al., 2020).

Type I PKSs of amphotericin synthesis genes were considered as core genes because these genes participate in the carbon skeleton of amphotericin, which is the rate-limiting step in amphotericin synthesis. The AmphA module of the amphotericin synthetic gene cluster synthetizes the first unit using acetyl-CoA as the precursor (Caffrey et al., 2001). However, a special phenomenon was found in which the expression level of the amphotericin synthesis gene cluster (except for AmphA) in high-yield *S. nodosus* ZJB2016050 was lower than that of wild-type *S. nodosus* ATCC14899, and the production of AmB of *S. nodosus* ZJB2016050 was higher than that of *S. nodosus* ATCC14899. In a fermentation experiment, we detected the total amount of AmA and AmB and found that wild-type *S. nodosus* ATCC14899 was higher than *S. nodosus* ZJB2016050 in the synthesis of amphotericin. The amphotericin skeleton of the wild-type strain preferred flowing to AmA rather than AmB. By comparing the total amount of amphotericin (the sum of AmA and AmB), we speculate that the total synthesis of amphotericin in wild-type *S. nodosus* ATCC14899 was more than that of *S. nodosus* ZJB2016050, which is due to the expression difference of the amphotericin synthesis gene cluster. The reason why high-yield strain prefers AmB is because the intracellular reduction (NADPH/NADP^+^) of *S. nodosus* ZJB2016050 was weaker than that of the wild type, which affected the reduction reaction in the ER5 domain of AmphC. In this study, we analyzed the PPP pathway because we speculated that the synthesis of AmA had an important relationship with intracellular reduction. The decrease in the upstream flow from G6P to glucono-1,5-lactone-6P would affect the whole subsequent step. Even if the 6-phosphogluconate dehydrogenase gene was also upregulated, the overall accumulation of NADPH may be reduced. In another study, we measured the intracellular reduction (NADPH/NADP^+^) of two strains and found that the high-yield strain had less intracellular reduction than the wild-type strain. The ER5 domain of the AmphC module in amphotericin synthesis consumes NADPH to reduce the C28–C29 of amphotericin and product AmA (Huang et al., 2020).

In past studies on the synthesis of amphotericin B, strategies such as increasing the expression of synthetic gene clusters, knocking out competitive secondary metabolites synthetic gene clusters, and increasing the supply of precursors had been used, while ignoring the biosynthesis of by-product amphotericin A. This study provides new research content for the research on the synthesis of amphotericin by *S. nodosus* and provides theoretical guidance for subsequent strain transformation.

## Conclusions

In summary, a mutated high-yield strain *S. nodosus* ZJB2016050 was obtained in our lab and had greater advantages in industrial production. By comparative transcriptome analysis, it was found that the expression of glucokinase and phosphoglycerate mutase was upregulated and promoted the utilization of glucose. Upregulation of pyruvate dehydrogenase and downregulation of aldehyde dehydrogenase contribute to the accumulation of acetyl-CoA. The expression differences of genes in the TCA cycle play a positive role in the accumulation of succinyl-CoA. A PKS type II synthesis gene cluster is downregulated in *S. nodosus* ZJB2016050. The total synthesis of amphotericin in wild-type *S. nodosus* ATCC14899 was more than that of *S. nodosus* ZJB2016050, which is due to the expression difference of the amphotericin synthesis gene cluster. The reason why the high-yield strain prefers AmB is because the intracellular reduction (NADPH/NADP^+^) of *S. nodosus* ZJB2016050 was weaker than that of the wild type, which affected the reduction reaction in the ER5 domain of AmphC.

## Data Availability Statement

The raw data supporting the conclusions of this article will be made available by the authors, without undue reservation.

## Author Contributions

KH and BZ conceived and designed the research. KH and YC conducted the experiments. Z-QL and Y-GZ contributed reagents or analytical tools. KH analyzed the data. KH and Z-QL wrote and modified the manuscript. All authors contributed to the article and approved the submitted version.

## Conflict of Interest

The authors declare that the research was conducted in the absence of any commercial or financial relationships that could be construed as a potential conflict of interest.
