# Supplementary material for: Comparative Transcriptome Analysis of Streptomyces nodosus Mutant With a High-Yield Amphotericin B
Source: Front Bioeng Biotechnol. 2021 Feb 1;8:621431. doi: 10.3389/fbioe.2020.621431 (PMC7882699; doi:10.3389/fbioe.2020.621431)
Supplement: Supplementary file 1 [file Data_Sheet_1.docx]

**Comparative transcriptome analysis of *Streptomyces nodosus* mutant with a high-yield amphotericin B**

Kai Huang^1,2^, Bo Zhang^1,2^, Yu Chen^1,2^, Zhi-Qiang Liu*^,1,2^, Yu-Guo Zheng^1,2^

*^1^The National and Local Joint Engineering Research Center for Biomanufacturing of Chiral Chemicals, Zhejiang University of Technology, Hangzhou 310014, P. R. China*

*^2^Key Laboratory of Bioorganic Synthesis of Zhejiang Province, College of Biotechnology and Bioengineering, Zhejiang University of Technology, Hangzhou 310014, China*

**Correspondence: Professor Zhi-Qiang Liu, College of Biotechnology and Bioengineering, Zhejiang University of Technology, Hangzhou 310014, China. Email: microliu@zjut.edu.cn; Tel: +86-571-88320614; Fax: +86-571-88320630*

**Table S1** Characterization of different mutagenesis colonies.

| Type | Photo | color | shape | spore | Positive mutation rate(%) |
| --- | --- | --- | --- | --- | --- |
| 1 | 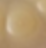 | yellowish | deplanate | asporous | 11.3 |
| 2 | 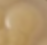 | yellow | convex | black spores | 15.8 |
| 3 | 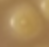 | yellowwish, | convex | gray spores | 72.6 |

**Supplement figures and captions：**

**Fig.S1** The morphology of *S. nodosus* ZJB2016050 and *S. nodosus* ATCC14899 shake flask cultures is in seed medium. Section **Results and Discussion 3.1 Mutagenesis results and morphological observation**.

**Fig. S2** Agar diffusion assays screening method for high-yield AmB *S. nodosus* screening. Section **Results and Discussion 3.1 Mutagenesis results and morphological observation**.

**Fig. S3** The AmA and AmB fermentation yield of *S. nodosus* ZJB2016050 and *S. nodosus* ATCC14899 at 96 h. Section **Results and Discussion 3.2 Electron microscopy and fermentation analysis**.

**Fig. S4** The distribution of base content for samples of *S. nodosus* ZJB2016050 and *S. nodosus* ATCC14899. Section **Results and Discussion 3.3 Analysis of the transcriptome reads of *S. nodosus***.

**Fig. S5** The base mass distribution of samples of *S. nodosus* ZJB2016050 and *S. nodosus* ATCC14899. Section **Results and Discussion 3.3 Analysis of the transcriptome reads of *S. nodosus***.

**Fig. S6** The composition profile of total raw reads for samples of *S. nodosus* ZJB2016050 and *S. nodosus* ATCC14899. Section **Results and Discussion 3.3 Analysis of the transcriptome reads of *S. nodosus***.

**Fig. S7** The homogeneity of sequencing results for samples of *S. nodosus* ZJB2016050 and *S. nodosus* ATCC14899. Section **Results and Discussion 3.3 Analysis of the transcriptome reads of *S. nodosus***.

**Fig. S8** The number of common genes and unique genes of samples *S. nodosus* ATCC14899 and *S. nodosus* ZJB2016050. The purple circle indicates sample *S. nodosus* ZJB2016050, and the pink circle indicates sample *S. nodosus* ATCC14899. Section **Results and Discussion 3.3 Analysis of the transcriptome reads of *S. nodosus***.

**Fig. S9** Volcano map of differentially expressed genes of sample *S. nodosus* ZJB2016050 conpared with sample *S. nodosus* ATCC14899**.** The up-regulated genes indicated by blue dots and the down-regulated genes indicated by red dots. Section **Results and Discussion 3.3 Analysis of the transcriptome reads of *S. nodosus***.

**Fig. S10**  Quantitative RT-PCR study of the expression levels of the 25 key genes of *S.nodosus* ATCC14899 and *S.nodosus* ZJB2016050. **Section Multiple genes or metabolic pathways analysis The most significant difference expressions attributive metabolic pathway.**

Fig.S1


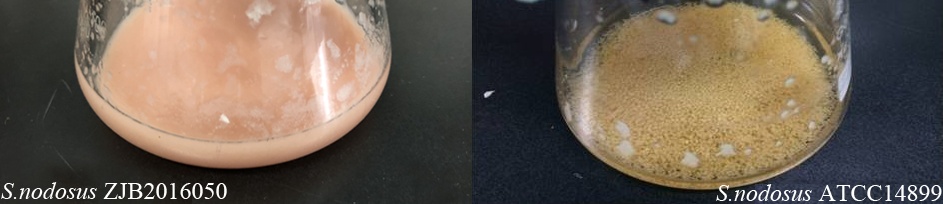


Fig.S2


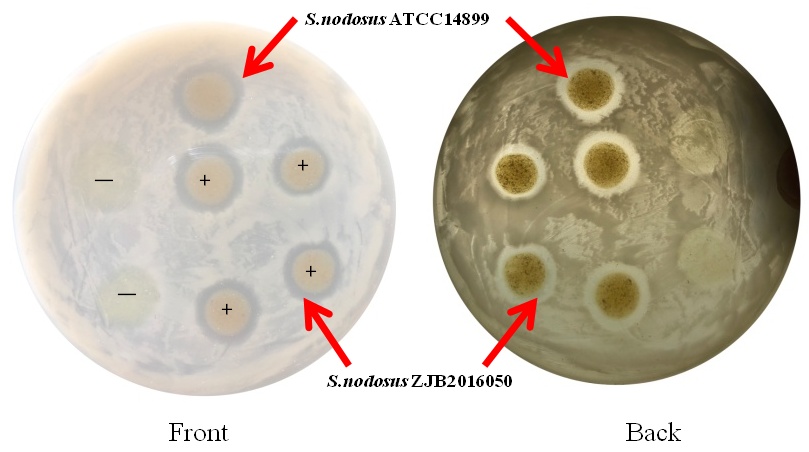


Fig.S3





Fig.S4


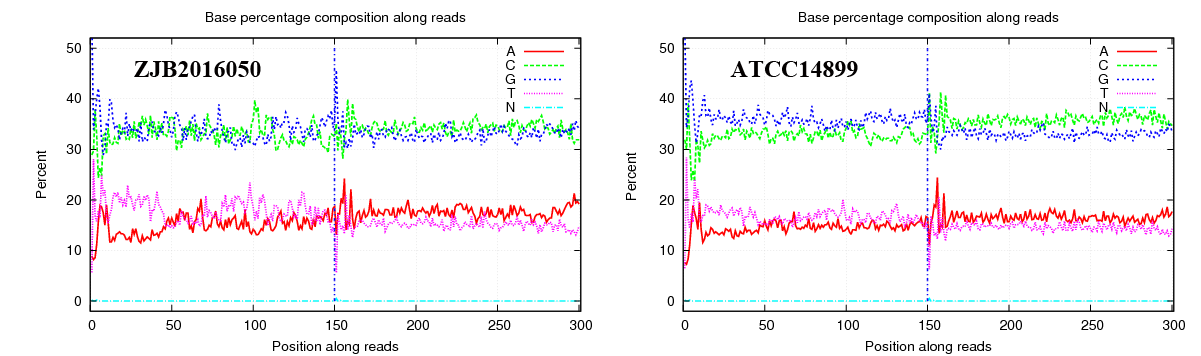


Fig.S5


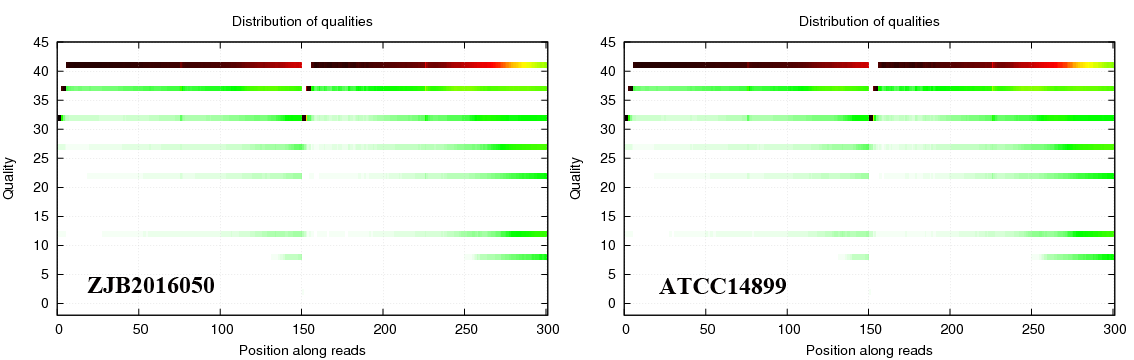


Fig.S6


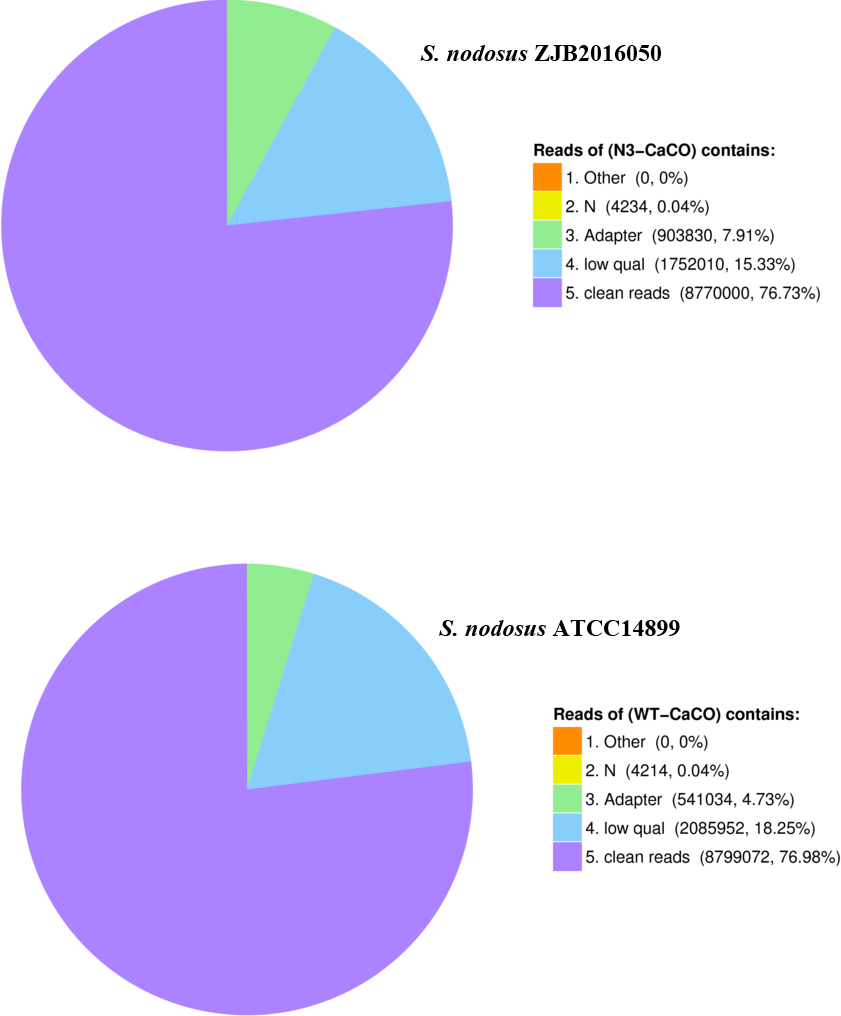


Fig.S7


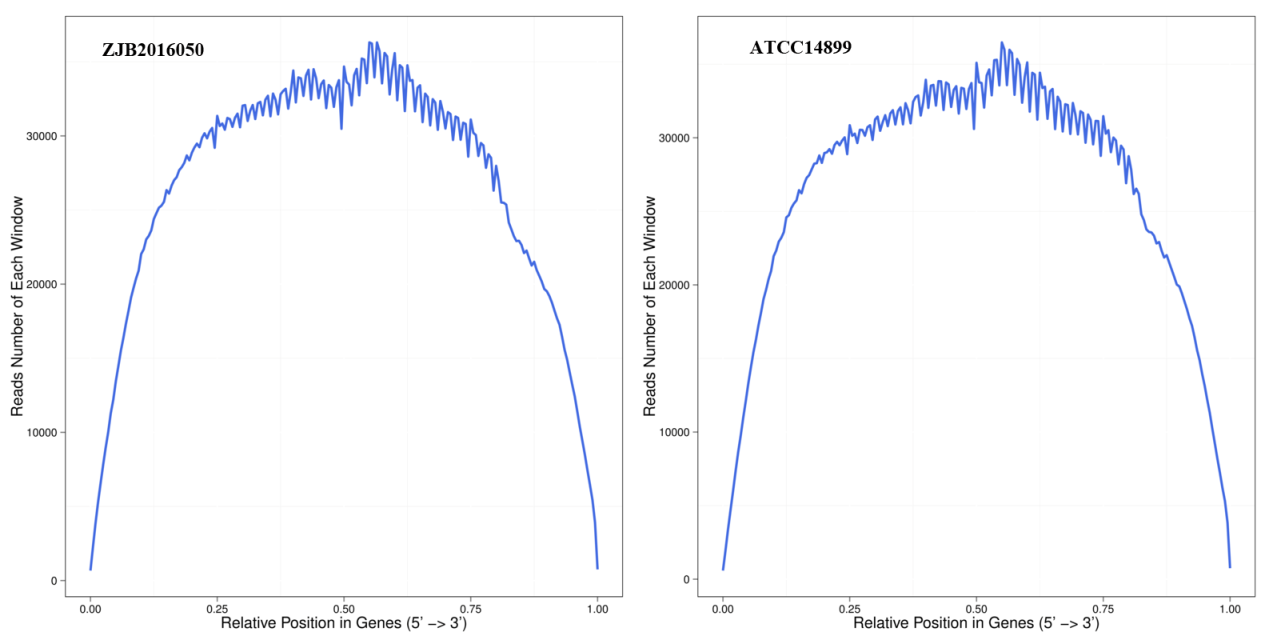


Fig. S8


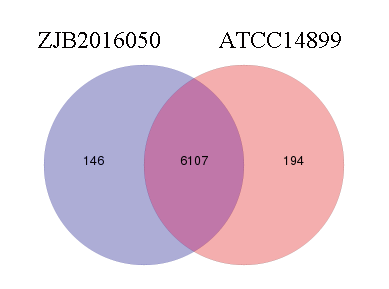


Fig. S9


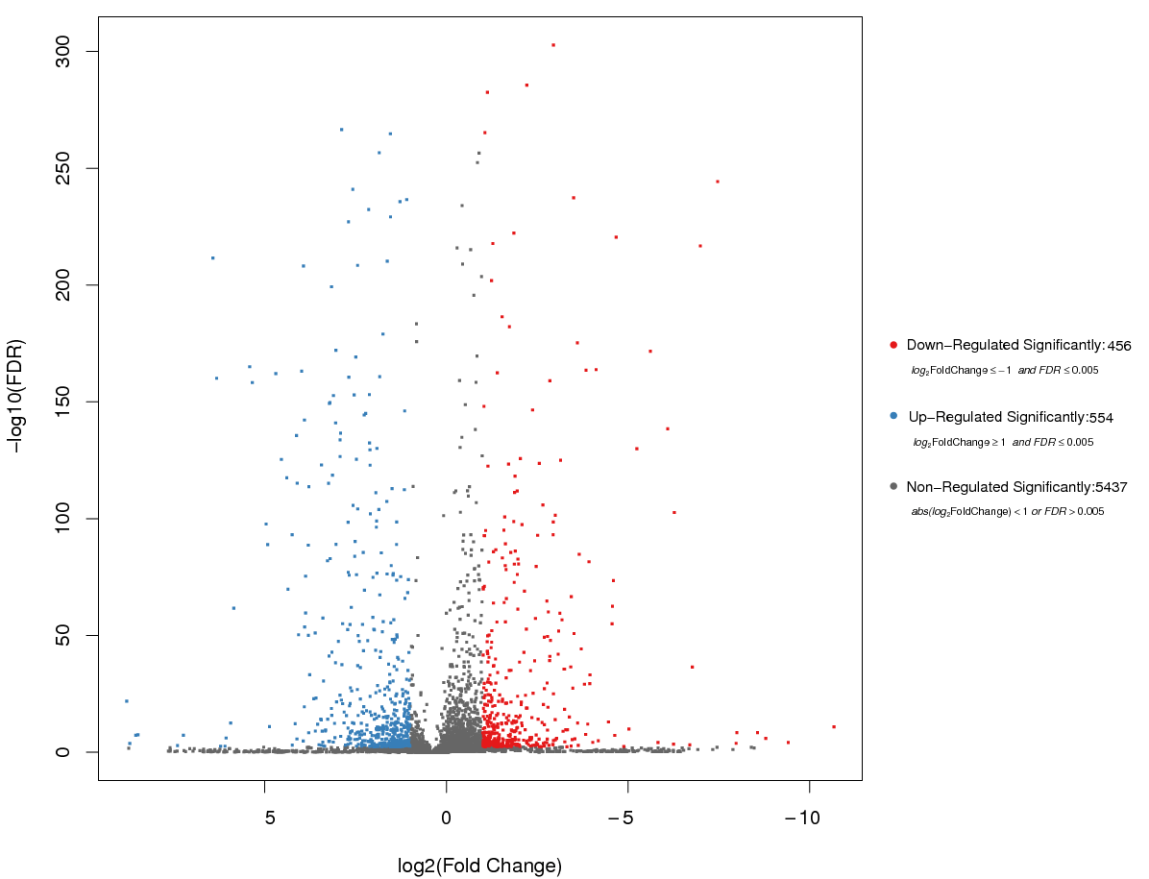


Fig.S10



**The sequence of unlabeled genes:**

Unlabeled gene 2:

GTGCCGCTGCGTCCGGGCGAGGGCGAGAAGGACCACCTCACCAGCCTGGAGGGCCTGGCCGCGCTGTCGCTGGACGCTCTCAGCTCGGTGGCGTACGGCCCCGAGGCGATCGTGCTGGCCCTGGTGGCAGCCGGCACCGGGGCGCTGACCGCGACCCTCCCCATCACGCTGGTGATCACCTTCCTGCTCGCGGTGCTCGTGGTGTCGTACGGCCAGGTGATCGCCGTGCACCCGGACGGGGGCGGGGCCTACGCCGTCTCCAAACGGGACCTGGGTCCCACCGTGAGTCTGCTCGCGGCGGCCAGCCTGGTCGTGGACTATGTGCTCACCGTCGCCGTCAGCCTCGCCGCGGGCTCCGACGCGCTTGCGTCCGCCTTCCCCTCCCTCGCCCATGACAAGCTCGTCATCTGCCTCATCGGACTGGCCCTGCTGACGGCCGTGAACCTACGGGGCATCACCGCCGGCGCCCGGGTGCTGATGCTGCCGACCGCGCTGTTCGTGGTGTCGGTGCTCGGCGTCGTCGTCCTAGGGCTGGCCCGCTCGCACCCGGCGGCCGTCGTGGGCACCCCGCAACCCCTCCACGCGCAGGAGACGCTGGGGATCCTGCTGATCCTCAAGGCGTTCTCCTCCGGCTGCTCGGCGCTGACCGGGGTGGAGGCCATCGCCAACGCCGTACCGACCTTCCGCGCCCCCCGGGTCAAGCGCGCCCAGCGCACCGAACTGATGCTGGGCGCGCTCCTCGCGCTGATGCTGATCGGCCTGGCGGTGCTCATCCGCCGTGACCATGTCGCCCCGCGCGGGAACGTCACGGTGCTGGCCCAGCTCACGGCCGGTGCCTTCGGCACCGGTTGGGCCTACTACGCCACCAACCTCGTCGTCACCCTGGCCCTGCTCCTCGCCGCCAACACCAGCTTCGGCGGACTGCCGGTGCTGATGAGCCTGCTCGCCCGGGACAACCGGCTGCCCCATCTGTTCGGGCTGCGCGCCGAACGCCCCGTCTACCGCCATGGCGTGGTGGCCCTGGGGCTGCTGGCCGCGGCGCTGCTGATCGCGGTCGACGCCGGCACGCACCGGCTGATCCCCTCTTCGCGATCGGTGTGTTCATCGGATTCACACTCAGTCAGCTGGGCCTGGTACGGCACTGGGCGACCGAGCGGCCGGGGCGATGGAGACACCGGGCACTGA

Unlabeled gene 3:

ATGTTGTTCGCCTATGAACTCCACCAGATCCGTTCCACCCAACTGATCCGTGAGGCGGAGCGCTTCCGGCTCCGCCGCGAGGTGCTGCGCGAGCGCCGCGCCGCCCGTAGGGTCCACGGAGAACCCGGAGCCGAGGGCGACGCCCCGCGCCGCTCCCGGAACCGGCCCCCCTTCGCCCGCGCCGCATGA

Unlabeled gene 4:

GTGGCGTCGGGGTGTCGTTGTGGGGGTGTGACACCTGGGGAGATGACCGAGGTCCGGGAGGACCTGGAGGCGTTCACGGCGGAGTTGTTCGACGGGTTCTTCCGCGCGGACCAGCGGCGCTGGGGGCAGGCGTATGTACGAGGGCTGCTGCTGGACGGGCGGCGCAAGTCGGTGGAACCGATGGCTGCCCGTCTCGGCGAGGACGGCAACCGCCAGGCGCTGGCGCACTTCATCACCTCCAGTCCGTGGAATGCGGCCCATGTGCGGGCCCGGCTGGCCTGGAGGATGTATGAAGCGATCGGTCCGGAGGCACTGATCGTCGACGACACCGGCTTCCTCAAGGACGGTAACGCCTCTGCATGCGTATCCCGGCAGTACACCGGCACCGCGGGCAAGGTCACCAACTGCCAGGTAGGCGTGTCGCTGCACCTGGCCAACGACCGTGCCTCGGCCGCGATCGACTGGCGGCTGTTCCTGCCCGCTTCCTGGGATCCCGCCTCGCCGGAGACGGATGCGGCCAAGGTCGCCCGCCGTCAGCGTTGCGGCGTCCCCGCCGACGCCGGCCATGTGGAGAAGTGGCAGTTGGCCCTGGACATGATCGACGAGGCCCGCAGTTGGGGCGTGGACGTTCCGCTGGTCGTCGCGGACGCCGGATACGGCGACGCCACCGCCTTCCGCCTGGCCCTGGAAGAACGGAAACTGGCTTACGCCGTCGGCGTCTCCTCCCGGCTCACTGCTCACCCTGAACATGCGCAGCCGGTTACCCCGCCTTATCAGGGCATTGGCCGACCGCCGGTGGCGACGTATCCGGACAAGCCGATGACGGTGAAGGAACTGGTCATCGAGGCCGGCCGGCAGGCGGCCCGGCCGGTGTCCTGGCGCGAGGGTTCCCGGCCGGGAAAGGGGCGCAGTGGCTTCAAACGCATGTACTCGCGCTTCGTCGTCCTGCGCATCCGGCCCGCCGGACGCGAGATCCGCCAGGCCGCCAAGGGCGCGGGGCTGCCCGAGCGATGGCTGCTGGCCGAATGGCCCGCCACCGAGCCGGAACCGGTGCAGTTCTGGCTGTCCAGTCTGCCCTCCGGCATGCCCCTGGCCTCGCTGGTCCGACTGGCCAAGCTCCGCTGGCGCATCGAGCACGACTACCGCGAGATGAAACAAGCCCTCGGACTGGCCCATTTCGAAGGCCGCACCTGGAACGGCTGGCACCACCACGTCACCCTCGTCTCGGCTGCCCACGCCTTCTGCACCCTGCAACGGCTGGCACACGACCCAAAAGACGCGGCGCAGGACTGA

Unlabeled gene 5:

ATGACCGGATCTGAGATTTTCCTCGCCAGCAAGCGCGCGGCGATCACCTACGACACCGACCCTGCCACCGGCGAACCTCGCGCCTGGCTGGCCCCTGGCGGCACCGGCAACGTTGTCGCCGAACAGGCCGGCGTCCTCAACATCTCCTGGATAGCCAGCGCCGACTCCGAGGACGACCGCCGTGCCTCGGCGCGCAACCCCGACGGCGTGACCATGGAACTGCATTCCGGCCGGGAGATCTTGGTCCGGCTCATCAGGCACGACCCCGCCGTCTTCCGTAACGTGCAGAACTTTATGACCGCCGACCTCATGTGGGCGGCCAACAACTACGGCTGGGACCGCTGGACCCAGCCGTCGTTCGGCGCCGACACCCGCGAAGGCTGGGCGGATTTCCGCCGCTTCACACGAGACTTCGCCGACGCGATCATAAAGAGCTCGGCCCAGTCACCGGACCCCGTCTACCTAGTCCATGACTACCAGCTGGTCGGGGTCCCCGCGCTGCTGCGCGAACAACGGCCGGACGCGCCGATCCTGGTCTTTGTGCACATCCCGTGGCCGTCGGCCGACTACTGGGAGGTGCTGCCCAAGGAGATCCGCACCGGCATTCTCCACGGTATGCTGCCCGCCACCACGATCGGCTTCTTCGCTGACCGTTGGTGCCGCAACTTCCTGGAGAGCGTGGCCGATCTTCTGCCCGACGCTCGGATTGACCGCGAGGCGATGACCGTCGAGTGGCGCGGCCACCGCACCCAATTGCGCACCATGCCGCTCGGCTACAGCCCGCTCACCCTGGAGGGCCGTGACCCGCGACTGCCCGACGGGATCGAGGAGTGGGCTGACGGCCACCGGCTGGTGGTGCACAGCGGGCGTACCGACCCGATAAAGAACGCCGAACGCGCGGTGCGCGCCTTCGTCCTGGCCGCCCGGAGCGGCGGGCTCGAGGACACCCGCATGCTGGTGCGGATGAATCCCAACCGACTGTACGTGCCGGCCAATGCCGACTACGTACACCGGGTGGAGACCGCCGTCGCCGAGGCCAACGCCGAGCTGGGCCCTGACACCGTGCGCATAGACAACGACAACGATGTGAACCACACCATCGCGTGCTTCCGCAGGGCCGACCTGCTCATCTTCAACTCAACCGTCGATGGTCAAAACCTCAGCACGTTCGAAGCGCCGCTGGTTAACGAACGCGACGCCGATGTGATCCTTTCGGAGACCTGCGGCGCAGCCGAGGTCCTGGGTGAGTACTGCCGCAGCGTCAACCCCTTCGATCTCGTCGAGCAGGCCGAGGCCATCTCCGCCGCGCTCGCTGCCGGGCCGCAGCAGCGGGCAGAGGCCGCCGCCCGCCGTCGCGATGCTGTCCGCCCCTGGACGCTCGAAGCCTGGGTGCAGGCCCAACTGGATGGACTCACTGCCGACCACGCGGCCCGCACGACAACCGCCGAGCGCCCCGACGCCGAGACGGCCGTCCCGGCACGAGCAGACCTCTGA

Unlabeled gene 6:

ATGATCGTCAACGAGCCCGTCCCGGACACCTTCGAGGACACTCCCGTGGCGGAGCGGGATCCGGAGTGGTTCAAGCGTGCCGTGTTCTACGAGGTCCTGGTGCGCTCGTTCCAGGACAGTGACGGGGACGGGGTGGGGGATCTGAAGGGGCTGACCGCGAAGCTGGACTATCTGCAGTGGCTGGGTGTCGACTGCCTGTGGCTGCCTCCCTTCTTCCGGTCGCCGCTGCGGGACGGCGGCTACGACGTGTCCGACTACACGGCGGTGCTGCCGGAGTTCGGGGATCTCGCGGATTTCGTGGAGTTCGTGGACGCCGCCCACCAGCGGGGCATGCGGGTGATCATCGATTTCGTGATGAACCACACCAGTGACCAGCATCCGTGGTTCCAGCAGTCCCGCACCGACCCGACCGGCCCCTACGGCGACTACTACGTGTGGGCGGACGACGACAAACAGTATGGGCAGGCGCGGGTCATCTTCGTGGACACCGAGGCCTCCAACTGGACGTTCGACCCGGTGCGCCAGCAGTACTACTGGCACCGTTTCTTCTCCCACCAGCCGGATCTGAACTACGACAACCCGGCCGTGCAGGAGGAGATCCTGGCCGCGTTGAGGTTCTGGCTCGATCTGGGCATCGACGGTTTCCGGCTGGACGCGGTCCCCTACCTCTACCAGCAGGAAGGCACCTGCTGCGAGAACCTGCCCGCCACCCACACCTTCCTCAAAAGGGTCCGCAAGGAGATCGACGCCCACTACCCCGACACCGTCCTGCTGGCGGAGGCCAACCAGTGGCCCGAGGACGTCGTCGACTACTTCGGCGACTATCCCAGCGGCGGCGACGAATGCCATATGGCGTTCCACTTCCCCGTCATGCCCCGCATCTTCATGGCCGTGCGCCGTGAATCGGCGCATCCCGTCTCGGAGATCCTCGCCAAGACCCCCCAGATCCCCGCCCACTGCCAGTGGGGCATCTTCCTGCGCAACCACGACGAGCTGACCCTGGAGATGGTCACCGACGAGGAACGCGACTACATGTGGGCCGAATACGCCAAAGACCCCCGCATGCGCGCCAACATCGGCATCCGCCGCCGTCTGGCACCGCTGCTGGACAACGACCGCAACCAGATCGAACTGTTCACCGCGCTGCTGCTGTCCCTGCCCGGCTCCCCGATCCTCTACTACGGAGACGAGATCGGCATGGGCGACAACATCTGGCTCGGCGACCGCGACGCCGTGCGCACACCGATGCAGTGGACCCCGGACCGCAACGCGGGTTTCTCCTCCTGCGACCCCGGCCGTCTCTACCTGCCCACGATCATGGACCCGGTCCACGGCTACCAGGTCACCAACGTCGAGGCCTCGATGTCCTCCCCGTCGTCCCTGCTGCACTGGACCCGCCGGATGATCGAGATCCGCAAACAGAACCCCGCGTTCGGGCTCGGCTCCTACACCGAGCTGGTCTCCTCCAACCCGGCCGTCCTGGCGTTCCTGCGTGAGTACAAGGACGACCTGGTGATGTGTGTCCACAACTTCTCCCGTTTCGCCCAGCCCACCGAACTCGACCTGACCCGCTTCCACGGCCGCCACCCCGTCGAACTCATCGGCCAGGTCCGCTTCCCACCCATCGGCGACCTCCCCTACCTCCTCACCCTCGCCGGCCACGGCTTCTACTGGTTCCGACTCCGCAAGGACGCCTCCCCGGCCACCAGGGTGAATGTGTTCGTGGGCAGCTGA

Unlabeled gene 7:

ATGCCTGGCCGGGTCCTGGTCATCGGCTCCGGCTACCTCGCTGGCCACATCGCCGCTCGTCTCACCGGGCTCGGCGTTGAGACGGTGCTCAGCTCTCGGGGTGCCCCGGTCATTCCTGAAAGCCGTGGTGTGCGCTGGACTCGGGTCGACGTCACGTCGGGACCACAGGTGGCTGCTCTGATGGATGCCGTCCAGCCCGACGCCGTTGTCGCCGTGCACGGCCCGTCCGACATCACCTGGTGCGAGGCGCATCCCGAAGAGGCGTACGCCACCCACCATGGCGGCGCCCGCAACATCGCGGCTGCACTGGACGGTCGGCCCGTCCTGCTGGTGTCGACCGACAACGTCTTCCCCGGCAAGGCGGAGAGCCATGGCGAGTCGGCGCAGCCCTTCCCGGCCAATGCCTACGGCCGGGCGAAGCTCGCGGCTGAGCAGGAACTGCTCGCCACCTCCTCTGCCCTCATCCTGCGGGTGAGTCTGGTCTACGGTTGGGAGGACTGCGGCCCGCGGCCCAACTTCCTCACCAGCGTTACTCGTTCGCTGATGCGTGGGGAGCAGCTGCGGATCCCCGCTGACCACTGGAACACGCCCGTTCTTGTGGAGGACGTGGCCGCGTGGGTGACGACGCTGATGAGTTCCGGCCGTACCGGGATGCTGCACCTCGGCGGGCCACGTCGCATCGGCCGAGTCGACTGGGCCAGGCACATCGCCCAGCAGATCGGTGCAGACCCCACGCTCATTGTGCCGACGCCGCGTGTCGGTACGGCCTATGCCTGTCGGCCGCGCAATGCCTGTCTGCACAGCGAGGTGGCCGCGGAGCTGCCCGAACTACAGCGCCACCGTCCGGTCGATGTTCTCGAAGCCACTCATGCCCTGATTTCCTGA

Unlabeled gene 8:

GTGACGGCACTCATCCCCGCGCCGTGTGACCTCGTGGTGGCCGACCTCGGTGGTACGACCCTGCGCGTGGGCCGTATCGGGGCGGGCACGTCCGAGGTGCGCGATGTGCAGCGGGTGCCTACCGACGGACTCGGTCGGTACGGGGCACTCGCCCCGCAGGAACTCCAGGACCGGGTCGTGGAGCAGCTCGGGCGGGAGATCGCGGCTCGCCTCGCCTGTCCGGGGCAACCACCTGCTCAGGCCGTGGCAGTGTCCTTCGCTGGGCCAATGACGTCCGACGGAGTCGTGCTTGCTGGCCCGACACTGTGGGGAGGACCTGCGGCACCACTGCCCATCGCGGACGTGTTGGCGAAGCATCTGGGCCTGCCAGTGGTAGCGGCCAACGACGTGACCGCAGCGGCCTGGCGCTACGCCTCGGCCGAGTCCGAGCCCTTCTGCCTGACCACTGTGAGCTCGGGCATCGGGAACAAGGTGTTCCGCCACGGCGAGATCGTCATCGACGAGCACGGGTACGGCGGGGAGATCGGCCACTGGCTCGTCGACCCCGCCCAGGACGCCGCGCCGTGCGAGTGCGGAGGACGCGGCCACCTCGGGGCGATCGCGTCAGGACGCGGCGCGCTATTCGCGGTGCGCGCGGCGGCGGCAGCCGACACCGTTGCCTTCGCGCGCTCGGCGTTGGCCGAGCCATCGGGCGGCGTGCCCGACGGCATCACCAACGAGGCGTTCGCGGCGGCGGCCCGAGCAGGCGACAGGTTCGCGCGGGAGTCGCTGCGCCGCTCACTGCAGCCCCTTGCGTCCGCGGTGAGCCTGCTCTTCACCGCGATCGGTGTGCGCCGCTACCTGTTCGTCGGGGGCTTCGCCCTGGCCTTGGGCGACACCTTCCTGACGCTGCTCGGCGACGAACTGGTGCGGATCGGCTGCTTCGGCTTGGACGAGCGTGCGACGCGCGCCATGCTCGCGCTGGGCGAGGACGACGACGACCACTGCCTCATCGGCATCGGTCAGTTGGCGGCAGATCGCCTCGGCACTCCCGGGGCCCTCGAGGCGACGGCATGA

Unlabeled gene 9:

ATGCTGGTACGTGGTGAGGGTATCCGCGCCTGGGACGCCGAGGGGCGCGAGTTTCTCGACTGCGTTTCTGGCACCTTTAACCTGCTGCTCGGACACAACCACCCGGAGGTGATGGCCGCAGTACGGGAACAGACCGAGCGGCTCGTCTTCGCCAGTTCGTCCTTCCAGACCGAACCCACCAACCGGGTAGTCCAAGAGCTGGCTGCCATCAGCCCGCCCAACCTCACACGGATCAACCTGCGCAGTTCCAGTGGCTCCACCGCCAACGAAGGCGCCATCAAGATGGCCCAGCTCCATACCGGCCGTCGCGACGTCATCGTGCCTTTCCGCGCCCACCTCGGCCAGAGTCTCGCGACGGCGAGCCTCAACGGCACCACCAAAATGCGCGCACCGTTTCCGCACCGCTACCCCGGCAGCCTTCACGTGCCAGGCCCCTACTGCTTCCGCTGCTTCTACCGCCAGACCCCCGAGAGTTGCGGCATGCTCTGCGTCGACCGCATCGAGGACTTCATCACCTACGCTAGCTCCGGCAGCGTGGCCTGCGTGGTGATCGAGCCGATCAGCGGCGCTGGCGGCAACATCGTCCCGCCCGACGGCTACCTCCAAGAGTTGCGTCGGTTCTGCGACGATCGTGAGATCGTGCTCATCTTCGACGAGATCCAGACCGGGCTGGGCCGCACCGGACAAATGTTCGCCGCCGACCACTTCGGTGTCCAGCCGCACATGATGACCCTCGCGAAGGGACTCACCGGAAGCGGCCTCCCCATGGCCGCCATCCTCACCGAGGAACGCATGGCCGGCTGGGACCGTAGCCTGCACAGCTTCACCTACGGTAGCCACACCCTGTCGGCTGCGGCCGCCCTGGCCACCCTGGAAATCGTCCAGCGCCCCGGCTTCCTGGAGAACGTACGGGCCAGCGGCGACGTGCTCCTGGACCGGCTGCGGGACCTCCAGAAGGACAACCCCGTGATCGGCGACGTCCGAGGGGTCGGCCTGATGATCGGGGTCGAGCTGGTGGAGCCCGACGGCACCAAGGCCGTCGCCCGTGCCCACGCCTACCAAAGGTCGCTCCAGGACCACGGCATTCTCACCCGTGTCTCCGAGCACGGAGACGGCAGCACGATCGAACTGCGCCCACCTCTCATCCTTACACCTGCCGACGCCCACACGGTTGCTGACCGGTTCGGCGAGGCGCTGGAGGCCGTCGCATGA
